# Clinico-biological features of T-cell acute lymphoblastic leukemia with fusion proteins

**DOI:** 10.1038/s41408-022-00613-9

**Published:** 2022-01-26

**Authors:** Thomas Steimlé, Marie-Emilie Dourthe, Marion Alcantara, Aurore Touzart, Mathieu Simonin, Johanna Mondesir, Ludovic Lhermitte, Jonathan Bond, Carlos Graux, Nathalie Grardel, Jean-Michel Cayuela, Isabelle Arnoux, Virginie Gandemer, Marie Balsat, Norbert Vey, Elizabeth Macintyre, Norbert Ifrah, Hervé Dombret, Arnaud Petit, André Baruchel, Philippe Ruminy, Nicolas Boissel, Vahid Asnafi

**Affiliations:** 1 Université de Paris (Descartes), Institut Necker-Enfants Malades (INEM), Institut national de la santé et de la recherche médicale (Inserm) U1151, and Laboratory of Onco-Hematology, Assistance Publique-Hôpitaux de Paris, Hôpital Necker Enfants-Malades, Paris, France; 2 Department of Pediatric Hematology and Immunology, Robert Debré University Hospital (AP-HP), Université de Paris, Paris, France; 3 Center for Cancer Immunotherapy, INSERM U932, Institut Curie, PSL Research University, Paris, France; 4 Department of Pediatric Hematology and Oncology, Assistance Publique-Hôpitaux de Paris (AP-HP), GH HUEP, Armand Trousseau Hospital, Paris, France; 5 Sorbonne Universités, UPMC Univ Paris 06, UMRS 938, CDR Saint-Antoine, GRC n°07, GRC MyPAC, Paris, France; 6 Systems Biology Ireland, School of Medicine, University College Dublin, Dublin, Ireland; 7 Department of Hematology, Université catholique de Louvain, CHU UCL Namur - site Godinne, Yvoir, Belgium; 8 Laboratory of Hematology, CHRU Lille, Lille, France and U1172, INSERM, Lille, France; 9 Laboratory of Hematology and EA 3518 University Hospital Saint-Louis, AP-HP and Université de Paris, Paris, France; 10 Hematology Laboratory, Marseille University Hospital Timone, Marseille, France; 11 Department of Pediatric Hematology and Oncology, University Hospital of Rennes, Rennes, France; 12 Service d’hématologie clinique, Hôpital Lyon Sud, Marseille, France; 13 Aix-Marseille Univ, Inserm, CNRS, Institut Paoli-Calmettes, CRCM, Marseille, France; 14 PRES LUNAM, CHU Angers service des Maladies du Sang et CRCINA INSERM, Angers, France; 15 Institut de Recherche Saint-Louis, Université de Paris, EA-3518 Paris, France; 16 Inserm U1245, Centre Henri Becquerel, Université de Rouen, IRIB, Rouen, France; 17 AP-HP, Hôpital Saint Louis, Unité d’Hématologie Adolescents et Jeunes Adultes, Paris, France

**Keywords:** Acute lymphocytic leukaemia, Cancer genetics

## Abstract

T-cell acute lymphoblastic leukemias (T-ALL) represent 15% of pediatric and 25% of adult ALL. Since they have a particularly poor outcome in relapsed/refractory cases, identifying prognosis factors at diagnosis is crucial to adapting treatment for high-risk patients. Unlike acute myeloid leukemia and BCP ALL, chromosomal rearrangements leading to chimeric fusion-proteins with strong prognosis impact are sparsely reported in T-ALL. To address this issue an RT-MPLA assay was applied to a consecutive series of 522 adult and pediatric T-ALLs and identified a fusion transcript in 20% of cases. *PICALM-MLLT10* (4%, *n* = 23), *NUP214-ABL1* (3%, *n* = 19) and *SET-NUP214* (3%, *n* = 18) were the most frequent. The clinico-biological characteristics linked to fusion transcripts in a subset of 235 patients (138 adults in the GRAALL2003/05 trials and 97 children from the FRALLE2000 trial) were analyzed to identify their prognosis impact. Patients with HOXA trans-deregulated T-ALLs with MLLT10, KMT2A and SET fusion transcripts (17%, 39/235) had a worse prognosis with a 5-year EFS of 35.7% vs 63.7% (HR = 1.63; *p* = 0.04) and a trend for a higher cumulative incidence of relapse (5-year CIR = 45.7% vs 25.2%, HR = 1.6; *p* = 0.11). Fusion transcripts status in T-ALL can be robustly identified by RT-MLPA, facilitating risk adapted treatment strategies for high-risk patients.

## Introduction

T-cell acute lymphoblastic leukemia (T-ALL) is an aggressive hematological cancer arising from the transformation of T cell precursors arrested at specific stages of differentiation [1, 2]. T-ALL represent 15% of pediatric and 25% of adult ALL. Despite modern poly-chemotherapy protocols, followed by allogeneic hematopoietic stem cell transplantation (HSCT) in high-risk cases, outcome of pediatric and adult patients with T-ALL remains poor, particularly in primary refractory and relapsed cases. After a transient initial response, about 20–30% of pediatric [3–5] and 40% of adult T-ALL patients relapse [6] with a 5-years overall survival (OS) below 20%. Understanding the mechanisms leading to treatment failure is therefore crucial to identify patients at high risk and adapt treatment in order to improve long term prognosis. Cytogenetic and global transcriptomic analyses led to the classification of T-ALL into molecular subgroups characterized by the abnormal expression of specific transcription factors (TF) (TAL1; LMO1/2; TLX1/3; LYL1; HOXA; MEF2C) and their blocked differentiation at specific stages of maturation [1, 7, 8]. Aberrant TF expression can be due to their juxtaposition to T Cell Receptor (TCR) loci or to somatic mutations in their enhancer regions. A number of additional, recurrent genetic abnormalities are found, including the loss of major tumor suppressive pathways (e.g., inactivating mutations of PTEN and of the CDKN2A tumor suppressor locus) and activation of oncogenic pathways (e.g., activating mutations in NOTCH1/FBXW7, IL7R/JAK pathway, epigenetic regulators, cell cycle, PI3K, and RAS signaling) [9–12]. Acquisition and selection of these molecular alterations entails the complex clonal evolution at the cellular level that occurs during T-ALL progression. Unlike acute myeloid leukemia (AML) and B-Cell Precursor acute lymphoblastic leukemia (BCP-ALL), which harbor numerous translocations leading to chimeric protein fusions [13, 14] such abnormalities are more rarely reported in large series of T-ALLs [15–20]. Recurrent chimeric protein fusions in T-ALL include rearrangements of *KMT2A* (*AFDN (AF6), MLLT1*, *ELL*), *SET-NUP214*, *ABL1 (NUP214-ABL1, BCR-ABL1)*, *MLLT10 (PICALM, DDX3X, NAP1L1, XPO1*), and the ETS family (*SPI* and *ETV6*). Given their individual low frequency, the clinico-biological features of T-ALLs harboring chimeric fusions within a comprehensive series remains elusive.

To address this issue, we designed and developed an RT-MPLA assay allowing identification of the majority of known fusion transcripts leading to chimeric proteins in T-ALLs. Applying this panel to a comprehensive, consecutive series of 522 adult and pediatric T-ALLs, we here report an unexpected overall incidence of 20% of fusion transcript. Their mutational landscape, associated clinico-biological features and prognostic impact on patients enrolled in the French GRAALL protocol for adult patients and the FRALLE 2000T protocol for pediatric patients are described.

## Methods

### T-ALL samples and clinical trials

Diagnostic peripheral blood (PB) or bone marrow (BM) samples from a consecutive series of 522 T-ALL patients, with RNA available, 206 children and 316 adults, were screened for fusion transcripts (fig. S1). Sample collection and analyses were obtained with informed consent in accordance with the Declaration of Helsinki with approval from the institutional review boards of institutions that participated in this study. Diagnosis of T-ALL was based on the World Health Organization 2008 criteria revised in 2016, defined by expression of cytoplasmic and/or surface CD3, and negativity of CD19 and MPO [21].

Adult patients aged from 16 to 59 years (*n* = 138) were included in the GRAALL03/05 trials (GRAALL-2003, #NCT00222027; GRAALL-2005, #NCT00327678) and pediatric patients aged from 1 to 19 years (*n* = 97) were treated according to FRALLE 2000 T guidelines in ten French centers members of the Société Française de lutte contre les Cancers et les leucémies de l’Enfant et de l’adolescent (SFCE) centers.

Definitions: Corticosensitivity was defined as circulating blasts <1 G/L on day 8. Complete remission (CR) was defined as: absence of physical signs of leukemia, BM with active hematopoiesis and <5% leukemic blast cells (identified morphologically), and normal cerebrospinal fluid.

### Biological analysis of T-ALL samples

Diagnostic PB or BM T-ALL samples were analyzed for immunophenotype and classified as follows: Immature T-ALL are TCR-and cTCRβ-. αβ-lineage T-ALL are TCRαβ or preαβ (TCR-) but with cTCRβ + . γδ-lineage T-ALL are TCRγδ, *SIL-TAL1* and *PICALM-MLLT10* detection was performed by RT-PCR as previously described [2, 22]). Oncogenic transcripts TLX1 and TLX3 quantification was performed by RQ-PCR (primer and probe sequences as previously described [23, 24]). Quantification of *HOXA9* expression was performed by qRT-PCR as previously described [25]. All methods are detailed in supplementary methods.

Diagnostic available DNA samples from 273 cases were also analyzed using an 80-gene pan-exon next-generation sequencing capture-panel (details included in supplementary methods and Table S1).

### RT-MLPA

Using previously published methods [26], we created a mix of 268 RT-MLPA probes to target 106 different genes (Table S2; Fig. S2). The procedure is detailed in supplementary data. All 141 5′ probes have a GTGCCAGCAAGATCCAATCTAGA tail at their 5′ ends and all 127 3′ probes a TCCAACCCTTAGGGAACCC tail at their 3′ ends to allow for final PCR amplification.

### Data analysis

Fisher’s exact and Mann–Whitney tests were used for clinical, biological and mutational analyses. Overall survival (OS) was calculated from the date of pre-phase initiation to the last follow-up date. The cumulative incidence of relapse (CIR) was calculated from CR to relapse date, censoring patients alive without relapse at the last follow-up date. The Event-Free-Survival was calculated from diagnosis date to first event among: induction failure, first hematologic relapse and death from any cause in first CR. Relapse and death in CR were considered as competitive events. Univariate analyses assessing the impact of categorical and continuous variables were performed with a Cox model. Statistical analyses were performed with STATA software (STATA 12.0 Corporation, College Station, TX) for survival and R software for others (v4.0.2). All *p* values were two-sided, with *p* < 0.05 denoting statistical significance.

## Results

### RT-MLPA detect an unexpected 20% incidence of fusion proteins in T-ALLs

**Fig. 1:**
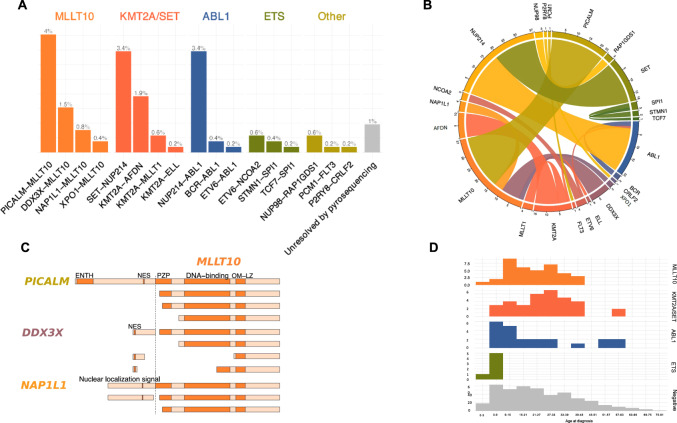
Identified fusion transcripts and incidence. **A** Histogram of identified transcripts, assigned to fusion groups. On top of each bars the percentage of all cases. *PICALM-MLLT10* (4%), *SET-NUP214* (3.4%), and *NUP214-ABL1* (3.4%) are the most recurrent. **B** Circos plot representing the fusion transcripts. **C** Schematic representation of *MLLT10* fused polypeptide chains with relevant domains. OM-LZ and nuclear addressing signals are always conserved. **D** Histograms of incidence of cases by age classes and fusion groups (absolute count on *y* axis). ETS fusion group is constituted with only pediatric cases. ENTH epsin N-terminal homology, NES nuclear export domain, PZP pregnancy zone protein.

A series of 522 T-ALL, all systematically screened for *PICALM-MLLT10*, *SET-NUP214,* and *NUP214-ABL1* by RT/qPCR, were evaluated for fusion transcripts by RT-MLPA. Fusion transcripts were detected in 104/522 (20%) cases (Table 1, S3A, B), leading to the identification of 99/104 fusion partners by pyrosequencing (Fig. 1A). Among the 5 unresolved cases 2 *PICALM-MLLT10*, 1 *NUP214-ABL1* and 1 *KMT2A-MLLT1* were identified by RT/qPCR. Only one t(11;16) involving *KMT2A* identified by cytogenetics and called “*KMT2A-?”* remained only partially identified. Importantly, among fusion transcripts systematically screened by RT/qPCR, no false negative or false positive RT-MLPA case was observed.

**Table 1:** Biological characteristics of T acute lymphoblastic leukemia according to fusion transcripts status.

RT-MLPA					Positive	Negative	All
					104	418	522
*Fusion groups*	*MLLT10* *n* = 37 (7%)	*KMT2A/SET n* = 34 (7%)	***ABL1*** *n* = 22 (4%)	*ETS* *n* = 6 (1%)	*Other* *n* = 5 (1%)	80%	
*Transcripts*	PICALM-MLLT10 (23) DDX3X-MLLT10 (8) NAP1L1-MLLT10 (4) XPO1-MLLT10 (2)	SET-NUP214 (18) KMT2A-AFDN (10) KMT2A-MLLT1 (4) KMT2A-ELL (1) KMT2A-? (1)	NUP214-ABL1 (19) BCR-ABL1 (2) ETV6-ABL1 (1)	ETV6-NCOA2 (3) STMN1-SPI1 (2) TCF7-SPI1 (1)	NUP98-RAP1GDS1 (3) PCM1-FLT3 (1) P2RY8-CRLF2 (1)		
*Population*							
Age mean (range)	23.4 (3–44)	27.9 (8–63)	23.1 (4–61)	**4.5 (1–8)**	18.8 (8–34)	25.9 (1–78)	25.4 (1–78)
Pediatric cases	41% (*n* = 15)	24% (*n* = 8)	55% (*n* = 12)	**100 % (*n*** = **6)**	60% (*n* = 3)	39% (*n* = 162)	39% (*n* = 206)
*Phenotype*							
Immature	3% (*n* = 1)	4% (*n* = 1)	7% (*n* = 1)	33% (*n* = 1)	33% (*n* = 1)	10% (*n* = 34)	9% (*n* = 39)
γδ-lineage	**84% (*n*** = **27)**	**81% (*n*** = **22)**	27% (*n* = 4)	0	33% (*n* = 1)	26% (*n* = 85)	34% (*n* = 139)
αβ-lineage	**12% (*n*** = **4)**	**15% (*n*** = **4)**	67% (*n* = 10)	67 % (*n* = 2)	33% (*n* = 1)	64% (*n* = 214)	57% (*n* = 235)
Not available	14% (*n* = 5)	21% (*n* = 7)	32% (*n* = 7)	50% (*n* = 3)	40% (*n* = 2)	20% (*n* = 85)	21% (*n* = 109)
*Homeobox Genes Deregulation*							
HOXA9	**95% (*n*** = **35)**	**100% (*n*** = **34)**	5% (*n* = 1)	0	60% (*n* = 3)	20% (*n* = 82)	30% (*n* = 155)
TLX1	0	0	14% (*n* = 3)	0	0	14% (*n* = 60)	12% (*n* = 63)
TLX3	0	0	**73% (*n*** = **16)**	0	0	14% (*n* = 57)	14% (*n* = 73)
Negative	**5% (*n*** = **2)**	0	**14% (*n*** = **3)**	**100% (*n*** = **6)**	40% (*n* = 2)	55% (*n* = 231)	47% (*n* = 244)

All means are compared against the Negative group with Wilcoxon test or Student test depending on the Shapiro test result. All proportions are compared against the Negative group with Pearson’s *χ*2 test or for small values with the Fisher exact test. Results with *p* ≤ 0.05 are displayed in bold. Immature T-ALL are TCR- and cTCRβ-. αβ-lineage T-ALL are TCRαβ or preαβ (TCR-) but with cTCRβ+ . γδ-lineage T-ALL are TCRγδ.

### Fusion transcripts are individually rare in T-ALL

**Fig. 2:**
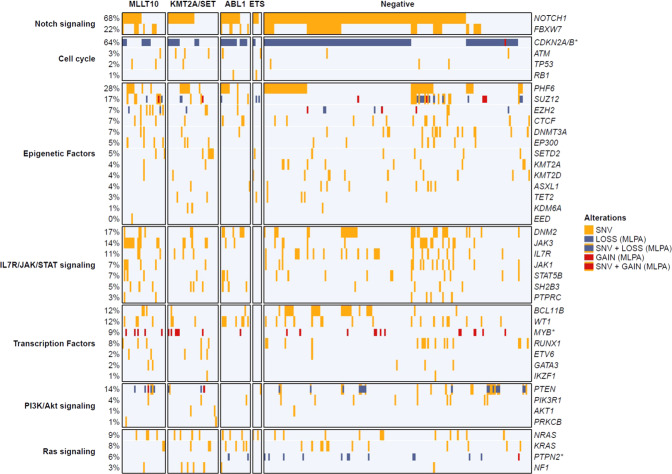
Mutational landscape according to fusion transcript group. Oncoplot depicting the genetic anomalies observed in each fusion transcript group. Genes are classified by functional groups. Each potent mutation is represented in yellow. Each line is a sequenced gene, each column a case.

Among identified transcripts *PICALM-MLLT10* (4%, *n* = 23), (Fig. 1A, B), *NUP214-ABL1* (3%, *n* = 19) and *SET-NUP214* (3%, *n* = 18) were the most frequently detected. All others fusion transcripts were rare (<2%). In order to facilitate analysis, RT-MPLA tested T-ALL were assigned into six groups: *MLLT10* recombinome (7%, *n* = 37) (Table 1); *KMT2A/SET* (grouped together due to their *HOXA* overexpression) (7%, *n* = 34); *ABL1* (4%, *n* = 22); *ETS* (1%, *n* = 6); other (1%, *n* = 5) and negative (80%, *n* = 418). Their available phenotype (*n* = 413), targeted DNA mutations (*n* = 273), *HOXA9* and *TLX1/3* overexpression data are resumed in Table 1 and Fig. 2.

### *MLLT10* recombinome

*MLLT10* recombinome was the most frequent chimeric protein (36% of identified transcripts). By order of occurrence *MLLT10* partners were *PICALM* (11q14, *n* = 23), *DDX3X* (Xp11, *n* = 8), *NAP1L1* (12q21, *n* = 4) and *XPO1* (2p15, *n* = 2) (Table 1). In keeping with the fact that OM-LZ domains are a key for immortalization [27], all *MLLT10* breakpoints conserved this domain (Fig. 1C).

The *MLLT10* cases were more frequently of TCRγ/δ lineage (*p* < 0.05) and *HOXA9* deregulated (Table 1). They were less likely to have *NOTCH1* signaling mutations (*p* = 0.03) and cell cycle mutation/deletion (*p* < 0.001) and showed an higher frequency of *RAS*, *IL7R/JAK/STAT* and epigenetic regulator mutations compared to negative samples (Table 2; Fig. 2).

**Table 2:** Genetic profile according to fusion transcripts status.

Fusion Group	Notch signaling	Cell cycle	Epigenetic factors	IL7R/JAK/STAT signaling	Transcription factors	PI3K/Akt signaling	Ras signaling	Total
MLLT10	**59% (*n*** = **17)**	**41% (*n*** = **12)**	76% (*n* = 22)	3% (*n* = 1)	52% (*n* = 15)	24% (*n* = 7)	24% (*n* = 7)	29
KMT2A/SET	**53% (*n*** = **18)**	**41% (*n*** = **14)**	59% (*n* = 20)	3% (*n* = 1)	35% (*n* = 12)	21% (*n* = 7)	32% (*n* = 11)	34
ABL1	75% (*n* = 15)	80% (*n* = 16)	60% (*n* = 12)	5% (*n* = 1)	45% (*n* = 9)	5% (*n* = 1)	**50% (*n*** = **10)**	20
ETS	67% (*n* = 4)	67% (*n* = 4)	67% (*n* = 4)	17% (*n* = 1)	17% (*n* = 1)	17% (*n* = 1)	33% (*n* = 2)	6
Negative	79% (*n* = 143)	76% (*n* = 136)	57% (*n* = 102)	1% (*n* = 1)	37% (*n* = 67)	19% (*n* = 35)	20% (*n* = 36)	180

All proportions are compared against the Negative group with Pearson’s *χ*2 test or for small values with the Fisher exact test. Results with *p* ≤ 0.05 are displayed in bold.

### *KMT2A* recombinome and *SET-NUP214* fusions

Seven percent of T-ALLs (34/522) (Table 1) demonstrated *KMT2A* or *SET* fusion transcripts, leading to *HOXA* overexpression without implication of *MLLT10*.

*SET-NUP214* gene fusions were identified in 18 cases, with uniform breakpoint positions. *KMT2A* 3′ partners were, by decreasing incidence, *AFDN* (*n* = 10), *MLLT1* (*n* = 4) and *ELL* (*n* = 1). As for the *MLLT10* group, and according to previous reports [20], significantly more of these T-ALL were from the γδ lineage (*p* < 0.05) and overexpressed *HOXA* transcripts (Table 1). They were significantly associated with less cell cycle genes mutations/deletion (*p* < 0.001) and fewer *NOTCH1* signaling genes mutations (53%, *p* = 0.002) compared to the negative group (Table 2; Fig. 2).

### *ABL1* recombinome

This group represented 4% of all T-ALLs and was dominated by *NUP214-ABL1* (19/22) transcripts. As expected [28], most of them were *TLX3* deregulated (*p* < 0.05), and a minority were *TLX1* deregulated [29]. This group harbored significantly more *RAS* signaling genes mutations/deletions (50%, *p* < 0.01) (Table 2; Fig. 2). Among the two *BCR-ABL1* transcripts, both were p190, one e6a2 and one e1a2.

### *ETS* family recombinome

*ETS* fusion transcripts were detected only in 1% of T-ALLs, all of which were pediatric cases, (Fig. 1D) with an incidence of 3% in the pediatric cohort. They included *ETV6-NCOA2* (*n* = 3), *STMN1-SPI1* (*n* = 2), and *TCF7-SPI1* (*n* = 1) transcripts. All *SPI1* breakpoints involved exon 3 [17] and all ETS domains were conserved in the fusion transcripts. No specific phenotype or transcription factor (*HOXA9*, *TLX1* nor *TLX3*) overexpression was observed within this subgroup (Table 1). Targeted sequencing identified significantly more cell cycle gene mutations (33%, *p* = 0.03) (Fig. 2; Table 2).

### Clinico-biological characteristics of fusion transcripts in GRAALL and FRALLE treated T-ALL

We then investigated the clinico-biological characteristics linked to fusion transcripts in a subset of 235 patients, including 138 adults enrolled in the GRAALL-2003/05 trials and 97 children enrolled in the FRALLE-2000T trial. A fusion transcript was observed in 27% of adults and 22% of children and the overall incidence of fusion transcripts in these cohorts was 25% (58/235).

**Fig. 3:**
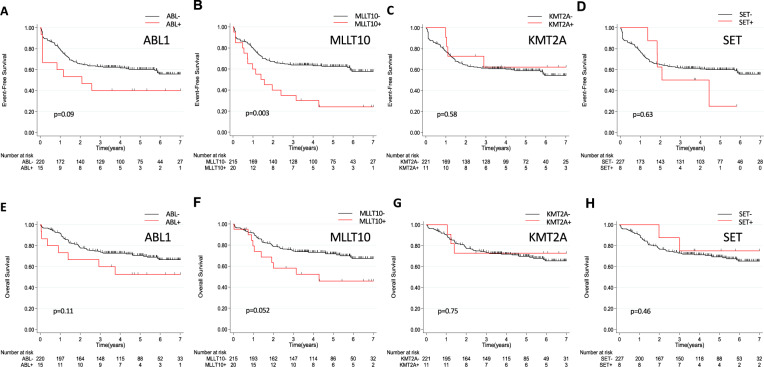
Clinical impact of the fusion transcripts group. **A**–**D** Event-Free Survival (EFS) and (**E**–**H**) Overall Survival (OS) according to fusion transcripts status in GRAALL and FRALLE treated patients.

The incidence of *ABL1* fusion was 6% (15/235). Patient outcome in this group did not differ significantly from negative cases (Table 3). Despite lower CR rates (66.7%) at the end of induction patients did not receive more frequent allogeneic HSCT and prognosis was not significantly (Table S4) different, with only a trend for shorter 5-year EFS (40% vs 60.2%, HR = 1.81; 95% CI [0.9–3.6]; *p* = 0.09) (Fig. 3).

**Table 3:** Clinical characteristics and treatment response in GRAALL and FRALLE treated patients according to fusion transcript status.

Fusion groups	Negative *n* = 177 (75%)	ABL1 *n* = 15 (6%)	KMT2A/SET *n* = 19 (8%)	MLLT10 *n* = 20 (9%)	ETS *n* = 2 (1%)	other *n* = 2 (1%)	All *n* = 235
Transcripts		*NUP214-ABL1* (14)	*SET-NUP214* (8)	*PICALM-MLLT10* (16)	*ETV6-NCOA2* (1)	*NUP98-RAP1GDS1* (2)	
		*BCR-ABL1* (1)	*KMT2A-AFDN* (5)	*NAP1L1-MLLT10* (1)	*TCF7-SPI1* (1)		
			*KMT2A-MLLT1* (4)	*DDX3X-MLLT10* (2)			
			*KMT2A-ELL* (1)	*XPO-MLLT10* (1)			
			*KMT2A-?* (1)				
*Clinical subsets*							
Age, median (range), y	19.9 (1.1–59)	15.9 (4.9–40.4)	27.5 (8.5–44.6)	**29.8 (5.8–45.6)**	4.6–4.2	12–12.8	21 (1.1–58.9)
WBC, median (range)	84.9 (2.8–788)	98 (12–980)	**29.2 (2.8–195)**	**28 (1.2–352)**	16–641	24.3–49	73.2 (1.2–980)
CNS involvement (%)	20 (11)	3 (20)	1 (5.3)	4 (20)	0	0	28 (11.9)
Male, n (%)	134 (76)	12 (80)	15 (79)	12 (60)	1 (50)	2 (100)	176 (74.9)
*Treatment response, n (%)*							
Poor steroid response	84 (47.4)	11 (73.3)	**16 (84.2)**	14 (70.0)	1 (50)	0	126 (53.6)
CR	161 (91)	**10 (66.7)**	19 (100)	17 (85.0)	2 (100)	2 (100)	211 (89.8)
MRD > 10^–4^	38 (29)	5 (55.6)	8 (53.3)	7 (58.3)	0	2 (100)	59 (34.3)
Allo HSCT	37 (22)	2 (20)	7 (36.8)	6 (35.3)	0	1 (50)	59 (28)

All subgroups characteristics are compared with the negative group. All results with *p* value ≤ 0.05 are displayed in bold.

*allo HSCT* allogeneic hematopoietic stem cell transplantation, *CNS* central nervous system, *CR* complete remission, *MRD* minimal residual disease, *WBC* white blood cell.

The incidence of HOXA *trans*-deregulated T-ALL with identified fusion transcripts was 17% (39/235) distributed as follows: *MLLT10* fusions in 20 patients (9%), *KMT2A* fusions in 11 patients (5%), *SET* fusions in 8 patients (3%). Compared to the negative group, patients with one of these 3 fusion transcripts were older with lower white blood cell counts (WBC). Overall, these patients have worse prognosis with a 5-year EFS of 35.7% vs 63.7% (HR = 1.63; 95% CI [1.02–2.6]; *p* = 0.04) and a trend for higher CIR (5-year CIR = 45.7% vs 25.2%, HR = 1.6; 95% CI [0.9–2.9]; *p* = 0.11) (Fig. S3). However, 5-year OS was not significantly different (59.3% vs 71.4%, HR1.26; 95% CI [0.7–2.2]; *p* = 0.43). This outcome cannot be explained by a significantly increased rate of allogeneic HSCT in this subgroup (36.1% vs 26.3%, *p* = 0.23).

When comparing each of these three fusions groups with their respective negative group, different patterns of treatment response and prognosis were identified. *KMT2A* fusions have no impact on treatment response and prognosis. On the contrary, patients with *SET* fusions were all corticosteroid-resistant with a higher rate of MRD positivity (71.4%) at the end of induction (data not shown) and higher 5-year CIR (75% vs 27.2%, *p* = 0.17). Despite good initial treatment response, patients with *MLLT10* fusions demonstrated a worse outcome, with shorter 5-year EFS (24% vs 62.4%, HR = 2.33; 95% CI [1.3–4.1]; *p* = 0.003) and shorter 5-year OS (45.7% vs 71.6%, HR = 1.94; 95% CI [0.99–3.8]; *p* = 0.052) (Fig. 3; table S4). This poor outcome remained on EFS and OS analysis of adult patients only (HR = 2.18; 95% CI [1.14–4.18]; *p* = 0.02 and HR = 2.19; 95%CI [1.07–4.51]; *p* = 0.03 respectively) (Fig. S4) and persists as a trend in children only in terms of EFS (HR = 2.81; 95% CI [0.99–8]; *p* = 0.052).

*ETS*-family (*n* = 2) and *NUP98* (*n* = 2) fusions were only identified in pediatric patients. Despite high MRD at the end of induction (>10^−4^) for both patients with *NUP98* rearrangements, only 1 patient required allogeneic HSCT. In contrast to previous report [17], all four patients had favorable outcome and remained in CR with a median follow up of 4.5 years (R, 3.4–6.7).

## Discussion

Last decade transcriptomic and genomic studies identified biological subgroups of T-ALL and uncovered major oncogenic and tumor suppressor pathways [1]. This molecular characterization provided a strong rationale for targeted therapies in T-ALL, such as drugs directed against JAK, NOTCH1, BCL-2 or PI3-AKT signaling pathways. However, contrary to BCP -ALL or AML, prognostic biomarkers identified in large prospective studies are yet lacking or debated to stratify patients at first line and adapt treatment.

In 522 consecutive adult and pediatric T-ALL RT-MLPA identified an unexpected 20% incidence of chimeric fusion protein. Fusion transcripts were correlated with their respective immunophenotypic, transcriptional and mutational landscapes, resulting in an unprecedented global overview.

*MLLT10* (10p12) (previously *AF10*) is a frequent 5′ and 3′ partner in chimeric fusion proteins harbored by T-ALL (8–10%) [30]. Published fusion partners include: *PICALM* (11q14) [31], *XPO1* (2p15) [32], *NAP1L1* (12q21) [11], *NUP98* (11p15) [33], *DDX3X* (Xp11) [19], and *HNRNPH1* (5q35) [19]. Within T-ALL, the most frequent 5′ partner is *PICALM*, with an incidence of 4–9% [2, 34, 35]. *MLLT10* translocations are associated with *HOXA* overexpression [36] and TCRγ/δ lineage orientation [2]. *MLLT10* contains a OM-LZ domain, known to bind to the epigenetic factor histone methyltransferase *DOT1L* [37, 38]. Direct fusion of *DOT1L* to *MLLT10* results in leukemic transformation and upregulation of *HOXA9* [37]. The outcome of cases harboring *MLLT10* fusion proteins is insufficiently described. Published studies are limited to children, with a trend for a pejorative outcome [35, 39]. In our series this unfavorable outcome was confirmed, with a shorter EFS and OS in the entire cohort and in the adult cohort highlighting the requirement alternative treatment in these cases. Same trends were observed in children but not reached significance because of low number of patients. Preclinical data showed an antiproliferative effect of demethylating agents via DOT1L in a model of transformed PICALM-MLLT10 cells [40].

Rearrangements involving *KMT2A* (11q23) [41] (previously *MLL*) are also recurrent in T-ALL with reported occurrence of 5–8% [7, 42]. Consistent with previous reports, the most frequent 3′ partners in our T-ALL cohort were: *AFDN* (6q27) [43] (previously *AF6*) and *MLLT1* (19p13, previously *ENL)* [44]. Of note, none of our 34 cases with *KMT2A* fusion demonstrated *KMT2A-AFF1 (*previously *MLL-AF4)*, or *KMT2A-MLLT3* (previously *MLL-AF9*) fusions, commonly observed in BCP-ALL and AML respectively. *KMT2A* rearranged T-ALLs over-express *HOXA* and are arrested in an early differentiation step after commitment to the γδ-lineage [1, 45]. The prognosis of *KMT2A-* rearrangements in T-ALL was unclear. Contrary to BCP-ALL, *KMT2A-MLLT1* in T-ALL has been reported to be favorable in children [46]. In keeping with this, in our series, patients with *KMT2A* rearrangements do not demonstrate worse outcome neither in terms of initial response to treatment neither in terms of relapse.

*SET-NUP214* (previously *TAF1-CAN*) is a recurrent chimeric protein found in 3–10% of T-ALLs [18, 47]. Most *SET-NUP214* chimeric proteins result from a cryptic deletion at chromosomic 9q34, leading to loss of one *ABL1* allele [48]. *SET-NUP214* induces *HOXA* deregulation by interacting with *XPO1*, *DOT1L* and the *HOXA* promoter [18, 49, 50]. As confirmed here, this recombination occurs in T-ALL from the γδ-lineage that are associated with a poor outcome [20]. Patients with SET-NUP214 chimeric protein have significantly poor initial treatment response and a trend for a higher risk of relapse without significant prognostic impact on EFS or OS. Thus, the need of intensifying treatment protocol in these patients is still matter of debate.

In T-ALL, *ABL1* (9q34) is predominantly rearranged with *NUP214* (4–6%, 9q34) by episomal amplification in association with *TLX1/3* deregulation [51, 52]. Other, rare reported, 3′ partners include *BCR* (22q11), *EML1* (14q32) [53], and *ETV6* (12p13) [54]. All confer constitutive activation of the tyrosine kinase domain of ABL1, with downstream LCK mediated proliferation [55] which could be targeted by tyrosine kinase inhibitors (TKI) such as dasatinib or bosutinib [56, 57]. The clinical impact of these translocations is debated, with a trend for a relatively pejorative impact [58]. In our series no prognosis impact of this rearrangement was demonstrated. However, due to this potentially targetable rearrangement these patients could benefit from the opportunity to add tyrosine kinase inhibitors to standard therapy [29].

The ETS family of proteins are TF containing an ETS DNA-binding domain [59] which are detected in pediatric T-ALL, fused in-frame to different 3′ or 5′ partners. *SPI1* (11p11.2 encoding PU.1) is fused in the 3′ position with *STMN1* (1p36) or *TCF7* (5q31) and *ETV6* (12p13) is fused in 5′ with *NCOA2* (8q13). *SPI1* fusions transcripts have been described in 4% of one pediatric T-ALL series, when they demonstrated a distinct gene expression profile and a poor outcome [17]. In contrast to this, all 4 FRALLE 2000T treated patients in our series had a favorable outcome.

RT-MLPA has the capacity to easily detect many rearrangements and its fast turnaround time and superior exhaustivity and reduced cDNA consumption could make it an interesting alternative to RT-PCR. Such a screening strategy will also identify patients with targetable rearrangement such as ABL1 [57] (with tyrosine kinase inhibitor) or KMT2A (with Dot1l or menin inhibitor) [40, 60] or who have poor prognosis and require treatment adaptation. However, RT-MLPA only allows detection of known fusion transcripts. New fusion transcripts detected by T-ALL RNA sequencing can easily be added to the RT-MLPA, thus providing a complement and extension to RNA-Seq approaches. In addition, the excellent specificity of this method was already demonstrated on a previous cohort of 540 patients with acute leukemia, confirming virtually all (98%) fusion transcripts detected by RT-PCR and Sanger sequencing [26]. As such, it is well adapted to routine clinical screening in Acute Leukemia.

## Supplementary information

Supplemental data
