# Supplementary material for: Clinico-biological features of T-cell acute lymphoblastic leukemia with fusion proteins
Source: Blood Cancer J. 2022 Jan 26;12(1):14. doi: 10.1038/s41408-022-00613-9 (PMC8791998; doi:10.1038/s41408-022-00613-9)

Supplemental data
Clinico-biological features of T-ALL with fusion proteins

1. Supplemental methods

Gene mutation screening

A custom capture Nextera XT gene panel (Illumina, San Diego, CA) targeting all coding exons and their adjacent splice junctions of 80 genes was designed, based on available evidence in hematological neoplasms. DNA Libraries were prepared using Nextera Rapid Capture Enrichment protocol and underwent 2x150bp paired-end sequencing on Illumina MiSeq sequencing system with MiSeq Reagent Kit v2 (Illumina). Briefly, sequence reads were filtered and mapped to the human genome (GRCh37/hg19) using in-house software (Polyweb, Institut Imagine, Paris). Annotated variants were selected after filtering out calls according to the following criteria: (i) coverage < 30x, <10 alternative reads or variant allelic fraction (VAF) <7%; (ii) Polymorphisms described in dbSNP, 1000Genomes, EVS, Gnomad and EXAC with a calculated mean population frequency >0.1%. Non-filtered variants were annotated using somatic database COSMIC (version 78) and ProteinPaint (St Jude Children's Research Hospital – Pediatric Cancer data portal), published data and in-silico prediction effect.

RT-MLPA

Method previously published [12]. All reactions were performed in a thermocycler with a heated lid (Mastercycler, Eppendorf, Hambourg, Germany). Total RNA (500 ng) was diluted in 4 µL of water. After the addition of 7.5 µL of a reverse transcription mix (2.5 µL 5x RT-MMLV Buffer (Invitrogen, Carlsbad, CA), 1 µL DTT 100 mM, 2 µL dNTPs 10 mM and 2 µL random

primers 100 µM), samples were heated for 1 min at 80 °C, incubated for 5 min at 37 °C and cooled at 4 °C. MMLV-Reverse transcriptase (1 µL) was added, and the samples were incubated for 15 min at 37 °C, heated for 2 min at 98 °C and cooled at 4 °C. cDNA (5 µL) was transferred to a new tube, and 3 µL of the LD-RTPCR probe mix (1.5 µL SALSA-MLPA Buffer (MRC-Holland, Amsterdam, The Netherlands) + 1.5 µL final dilution probe mix) were added. Samples were heated for 2 min at 95 °C and incubated for 1 h at 60 °C to allow the annealing of the LD-RTPCR probes. The ligation step was performed by cooling all samples at 54 °C and adding 32 µL of a ligation mix (3 µL SALSA-Ligase 65 Buffer A, 3 µL SALSA-Ligase Buffer B, 25 µL water, 1 µL SALSA-Ligase 65 (MRC-Holland)). This mix was incubated for 15 min at 54 °C and heated at 98 °C for 5 min to stop the ligation reaction.

For PCR amplification, 5 µL of the ligation products were transferred to new tubes containing 45 µL of a PCR mix (20 µL Red'y'Star Mix (Eurogentec, Liege, Belgium); 1 µL 10 µM 5' biotinylated primer U1 (GGGTTCCCTAAGGGTTGGA), 1 µL 10 µM primer U2 (GTGCCAGCAAGATCCAATCTAGA) and 18 µL water). Alternatively, a biotinylated primer U2 was used together with an unmodified primer U1 to allow the characterisation of the junction from the other end. The amplification was performed as follow: 6 min at 94 °C; 35 cycles (30 sec at 94 °C, 30 sec at 58 °C, 30 sec at 72 °C); 4 min at 72 °C; and cooled at 16 °C. PCR products (10 µL) were analysed on 8% acrylamide / bis-acrylamide (40/1) gels, and 20 µL were analysed using pyrosequencing to identify the two partner genes on a PyroMark Q24 platform (Qiagen, Venlo, the Netherlands) with a 15(ACTG) dispensation order following standard procedures.

2. Supplemental tables

Table S1. Custom capture Nextera XT gene panel

Custom capture Nextera XT gene panel					
AKT1	CTCF	HLA-B	MLL_KMT2A	PTPRD	STAT5B
ARID1A	CTNNB1	HNRNPA1B	MLL2_KMT2	RB1	SUZ12
ASB2	CXCR4	HRAS	NF1	RELN	TAL1
ASXL1	DDX3X	IDH1	NOTCH1	RHOA	TBL1XR1
ATM	DNM2	IDH2	NRAS	RPL10	TDRD6
BCL11B	DNMT3A	IKZF1	ODZ2	RPL5	TET1
CARD11	EED	IL7R	PHF6	RUNX1	TET2
CBL	EP300	IRF2BP2	PIAS1	SAMHD1	TP53
CBLB	ETV6	IRF4	PIK3CA	SETD2	TSR1
CCR4	EZH2	JAK1	PIK3R1	SF3B1	TYK2
CCR7	FAS	JAK3	PLCG1	SH2B3	UTX
CD58	FBXW7	JAKMIP2	POT1	SHP1	VAV1
CEBPA	FLT3	KIT	PRKCB	SKP2	WT1
CNOT3	FOXO3	KRAS	PTEN	SOCS1	ZEB1/TCF8
CREBPP	FYN	LCK	PTPN11	STAT1	ZFP36L2
CSNK1A1	GATA3	LEF1	PTPN2	STAT3	ZRSR2
CSNK2B	GPR183	MGA	PTPRC	STAT5A	
LMO1*	TAL1*	CD28	CISH	ZFHX4	
LMO2*	B2M	STIL	SOCS3		

Table S2. RT-MLPA probes

Gene	Exon	3'/5'	GSP	Probe
ATF7IP	13	5'	TCGGCAGGTCAATCCCCAAAATA	GTGCCAGCAAGATCCAATCTAGATCGGCAGGTCAATCCCCAAAATA
BCR	1	5'	GGCGCCTTCCATGGAGACGCAG	GTGCCAGCAAGATCCAATCTAGAGGCGCCTTCCATGGAGACGCAG
BCR	4	5'	GGGCGACCTCTTCCAGAAGCTG	GTGCCAGCAAGATCCAATCTAGAGGGCGACCTCTTCCAGAAGCTG
BCR	6	5'	ATCCAACGACCAAGAACTCTCTGGAAA	GTGCCAGCAAGATCCAATCTAGAATCCAACGACCAAGAACTCTCTGGAAA
BCR	13	5'	ATTCCGCTGACCATCAATAAGGAAG	GTGCCAGCAAGATCCAATCTAGAATTCCGCTGACCATCAATAAGGAAG
BCR	14	5'	AGCCACTGGATTTAAGCAGAGTTCAA	GTGCCAGCAAGATCCAATCTAGAAGCCACTGGATTTAAGCAGAGTTCAA
BCR	19	5'	ACTGAAGGCAGCCTTCGACGTCA	GTGCCAGCAAGATCCAATCTAGAACTGAAGGCAGCCTTCGACGTCA
BCR	7	5'	AGGAGCACGCTGGTCCTCCAT	GTGCCAGCAAGATCCAATCTAGAAGGAGCACGCTGGTCCTCCAT
BCR	12	5'	CATCCGGGAGCAGCAGAAGAAGT	GTGCCAGCAAGATCCAATCTAGACATCCGGGAGCAGCAGAAGAAGT
CCDC6	1	5'	TGCGCAAAGCCAGCGTGACCATC	GTGCCAGCAAGATCCAATCTAGATGCGCAAAGCCAGCGTGACCATC
CCDC6	7	5'	CGAGTTCAAGCAGGCCTATATCACCTG	GTGCCAGCAAGATCCAATCTAGACGAGTTCAAGCAGGCCTATATCACCTG
CNTRL	40	5'	CAGTATGAGTACACGGAGCTCAAGAAACAG	GTGCCAGCAAGATCCAATCTAGACAGTATGAGTACACGGAGCTCAAGAAACAG
CUX1	11	5'	GCGCCAAAAACAGCACACTCAAA	GTGCCAGCAAGATCCAATCTAGAGCGCCAAAAACAGCACACTCAAA

DDX3X	1	5'	CGCTCGGGCTGGACCAGCAG	GTGCCAGCAAGATCCAATCTAGACGCTCGGGCTGGACCAGCAG
DDX3X	2	5'	AGAGTGGAGGAAGTACAGCCAGCA	GTGCCAGCAAGATCCAATCTAGAAGAGTGGAGGAAGTACAGCCAGCA
DDX3X	3	5'	CTCATTTAAGGAACCGAGAAGCTACTAAAG	GTGCCAGCAAGATCCAATCTAGACTCATTTAAGGAACCGAGAAGCTACTAAAG
DDX3X	4	5'	GTGGAAGTGGATCAAGGGGAAG	GTGCCAGCAAGATCCAATCTAGAGTGGAAGTGGATCAAGGGGAAG
DDX3X	5	5'	CACCAAGTGAACGCTTGGAACA	GTGCCAGCAAGATCCAATCTAGACACCAAGTGAACGCTTGGAACA
DDX3X	6	5'	CAACAACTGTCCTCCACATATTGAAAGT	GTGCCAGCAAGATCCAATCTAGACAACAACTGTCCTCCACATATTGAAAGT
DDX3X	7	5'	TGATGGCTTGTGCCCAAACAG	GTGCCAGCAAGATCCAATCTAGATGATGGCTTGTGCCCAAACAG
DDX3X	8	5'	GAGGCTTTGAGGGCCATGAAG	GTGCCAGCAAGATCCAATCTAGAGAGGCTTTGAGGGCCATGAAG
DDX3X	9	5'	AGTACAGATCTACGAGGAAGCCAGAAAA	GTGCCAGCAAGATCCAATCTAGAAGTACAGATCTACGAGGAAGCCAGAAAA
DDX3X	10	5'	AAGAGGAAAGATTGGATTAGACTTTTGCAA	GTGCCAGCAAGATCCAATCTAGAAAGAGGAAAGATTGGATTAGACTTTTGCAA
EBF1	14	5'	CTGCCAACTCCCCCTATGCCA	GTGCCAGCAAGATCCAATCTAGACTGCCAACTCCCCCTATGCCA
EBF1	10	5'	AAGGAACACCAGGCAGATTCATTTATACAG	GTGCCAGCAAGATCCAATCTAGAAAGGAACACCAGGCAGATTCATTTATACAG
EBF1	15	5'	CCAACGGGAACAGCCTGCAAG	GTGCCAGCAAGATCCAATCTAGACCAACGGGAACAGCCTGCAAG
EBF1	13	5'	GCATCACAAGCCACCAATCAGG	GTGCCAGCAAGATCCAATCTAGAGCATCACAAGCCACCAATCAGG
EML1	17	5'	CAGCTCTCTGTAATGCGATACTCACCAG	GTGCCAGCAAGATCCAATCTAGACAGCTCTCTGTAATGCGATACTCACCAG
ETV6	4	5'	GGTCATACTGCATCAGAACCATGAAGAAG	GTGCCAGCAAGATCCAATCTAGAGGTCATACTGCATCAGAACCATGAAGAAG
ETV6	5	5'	CCATGCCCATTGGGAGAATAGCAG	GTGCCAGCAAGATCCAATCTAGACCATGCCCATTGGGAGAATAGCAG
ETV6	6	5'	GCTCGACTGTGGGGAAACCATAAG	GTGCCAGCAAGATCCAATCTAGAGCTCGACTGTGGGGAAACCATAAG
ETV6	7	5'	AGCCAGGACAAAGGCTTTTGTTCAG	GTGCCAGCAAGATCCAATCTAGAAGCCAGGACAAAGGCTTTTGTTCAG
CEP43	5	5'	AAGGGCCAACCACTGGGGAA	GTGCCAGCAAGATCCAATCTAGAAAGGGCCAACCACTGGGGAA
CEP43	6	5'	AAAACAAGTGCACAGACAACACCAAGTAAG	GTGCCAGCAAGATCCAATCTAGAAAAACAAGTGCACAGACAACACCAAGTAAG
CEP43	7	5'	CGGGCAGAAGGCTGGTGACAAG	GTGCCAGCAAGATCCAATCTAGACGGGCAGAAGGCTGGTGACAAG
FOXP1	19	5'	GCACACCTCTCAATGCAGCTTTACAG	GTGCCAGCAAGATCCAATCTAGAGCACACCTCTCAATGCAGCTTTACAG
HNRNPH1	3	5'	CAAGGAAGAAATTGTTCAGTTCTTCTCAG	GTGCCAGCAAGATCCAATCTAGACAAGGAAGAAATTGTTCAGTTCTTCTCAG
HNRNPH1	4	5'	GAAACACAAGGAAAGAATAGGGCACAG	GTGCCAGCAAGATCCAATCTAGAGAAACACAAGGAAAGAATAGGGCACAG
HNRNPH1	5	5'	GCGTGGTGCTTATGGTGGAG	GTGCCAGCAAGATCCAATCTAGAGCGTGGTGCTTATGGTGGAG
HNRNPH1	6	5'	GGATTTGGGTCAGATAGATTTGGAAGAG	GTGCCAGCAAGATCCAATCTAGAGGATTTGGGTCAGATAGATTTGGAAGAG
HNRNPH1	7	5'	TACAGAGCTACTGAGAATGACATTTATAAT	GTGCCAGCAAGATCCAATCTAGATACAGAGCTACTGAGAATGACATTTATAAT
HNRNPH1	8	5'	AGCTATGTCAAAAGACAAAGCAAATATGC	GTGCCAGCAAGATCCAATCTAGAAGCTATGTCAAAAGACAAAGCAAATATGC
HNRNPH1	9	5'	AGCAAGCGGTGGTGCTTACG	GTGCCAGCAAGATCCAATCTAGAAGCAAGCGGTGGTGCTTACG
HNRNPH1	10	5'	GATGGGAGGCATGGGCTTGT	GTGCCAGCAAGATCCAATCTAGAGATGGGAGGCATGGGCTTGT
HNRNPH1	11	5'	CCAGAGCAGCATGAGTGGATACG	GTGCCAGCAAGATCCAATCTAGACCAGAGCAGCATGAGTGGATACG

HNRNPH1	12	5'	CCAGTGATTTTCAATCAAACATTGCATAG	GTGCCAGCAAGATCCAATCTAGACCAGTGATTTTCAATCAAACATTGCATAG
INPP5D	9	5'	TGAGGACAAGTTCTACAGCCACAAGAAAA	GTGCCAGCAAGATCCAATCTAGATGAGGACAAGTTCTACAGCCACAAGAAAA
L3MBTL3	24	5'	GAAGAACATGGAAAGGTATTTAAAGATGAA	GTGCCAGCAAGATCCAATCTAGAGAAGAACATGGAAAGGTATTTAAAGATGAA
MEF2D	7	5'	CAGGAAAGGGGTTAATGCATCACTTG	GTGCCAGCAAGATCCAATCTAGACAGGAAAGGGGTTAATGCATCACTTG
KMT2A	9	5'	ATCCCTGTAAAACAAAAACCAAAAGAAAAG	GTGCCAGCAAGATCCAATCTAGAATCCCTGTAAAACAAAAACCAAAAGAAAAG
KMT2A	10	5'	CAGATGGATTTTACAGGATCAGAGTGGACTTTAAG	GTGCCAGCAAGATCCAATCTAGACAGATGGATTTTACAGGATCAGAGTGGACTTTAAG
KMT2A	11	5'	CTCTGTGCCAGTAGTGGGCATGTAGAG	GTGCCAGCAAGATCCAATCTAGACTCTGTGCCAGTAGTGGGCATGTAGAG
KMT2A	12	5'	GTGGAAGGCAACATCAGGCTACAAAG	GTGCCAGCAAGATCCAATCTAGAGTGGAAGGCAACATCAGGCTACAAAG
NAP1L1	5	5'	GCTGTTCTCTATCAGCCTCTATTTGATAAG	GTGCCAGCAAGATCCAATCTAGAGCTGTTCTCTATCAGCCTCTATTTGATAAG
NAP1L1	6	5'	GAAACCAGATGAAGAAGATGAGATTTCG	GTGCCAGCAAGATCCAATCTAGAGAAACCAGATGAAGAAGATGAGATTTCG
NAP1L1	7	5'	ATGTTGACTTGCTCAGTGATATGGTTCAG	GTGCCAGCAAGATCCAATCTAGAATGTTGACTTGCTCAGTGATATGGTTCAG
NAP1L1	8	5'	CTCAGATGCTGGCCAGCCTATG	GTGCCAGCAAGATCCAATCTAGACTCAGATGCTGGCCAGCCTATG
NAP1L1	9	5'	GGACCAGAAATTATGGGTTGTACAGG	GTGCCAGCAAGATCCAATCTAGAGGACCAGAAATTATGGGTTGTACAGG
NAP1L1	10	5'	TCTTTAACTTTTTTGCCCCTCCTGAAG	GTGCCAGCAAGATCCAATCTAGATCTTTAACTTTTTTGCCCCTCCTGAAG
NAP1L1	11	5'	CTGAAGTTCCTGAGAGTGGAGATCTG	GTGCCAGCAAGATCCAATCTAGACTGAAGTTCCTGAGAGTGGAGATCTG
NAP1L1	12	5'	GGAGAAGCTATTGAAGATGATGATGATGAT	GTGCCAGCAAGATCCAATCTAGAGGAGAAGCTATTGAAGATGATGATGATGAT
NAP1L1	13	5'	GAAGAAGGTGAAGAAGCGGATGAG	GTGCCAGCAAGATCCAATCTAGAGAAGAAGGTGAAGAAGCGGATGAG
NAP1L1	14	5'	GAAAATGATCCAGACTATGACCCAAAG	GTGCCAGCAAGATCCAATCTAGAGAAAATGATCCAGACTATGACCCAAAG
NCOR1	34	5'	CGACCTCTACCTGCGGCCAG	GTGCCAGCAAGATCCAATCTAGACGACCTCTACCTGCGGCCAG
NDE1	6	5'	GTTCAGAGACTGAAGGATGAAGCCAGAG	GTGCCAGCAAGATCCAATCTAGAGTTCAGAGACTGAAGGATGAAGCCAGAG
NUP214	26	5'	TCAGTTATCATCTGGTGACAAAGCTTCAG	GTGCCAGCAAGATCCAATCTAGATCAGTTATCATCTGGTGACAAAGCTTCAG
NUP214	29	5'	GGTTCAGCTTTTGCCAAGCTTCAG	GTGCCAGCAAGATCCAATCTAGAGGTTCAGCTTTTGCCAAGCTTCAG
NUP214	32	5'	TGGGTTTTCCTCTCCAAACAAAACAG	GTGCCAGCAAGATCCAATCTAGATGGGTTTTCCTCTCCAAACAAAACAG
NUP214	23	5'	GCAGATGGCCAGTCAGGCACCAG	GTGCCAGCAAGATCCAATCTAGAGCAGATGGCCAGTCAGGCACCAG
NUP214	28	5'	CGTCTTCTAATTTCACTGCTGCACAAG	GTGCCAGCAAGATCCAATCTAGACGTCTTCTAATTTCACTGCTGCACAAG
NUP214	31	5'	GGCTTTGGATCCACAGCTACCTCAA	GTGCCAGCAAGATCCAATCTAGAGGCTTTGGATCCACAGCTACCTCAA
NUP214	34	5'	TGGTTTTGGATCAGGCACAGGAG	GTGCCAGCAAGATCCAATCTAGATGGTTTTGGATCAGGCACAGGAG
NUP214	30	5'	AGTACTGGTGGAATAGTCTTTGGCCAG	GTGCCAGCAAGATCCAATCTAGAAGTACTGGTGGAATAGTCTTTGGCCAG
NUP98	12	5'	GGAGCCCCCCAGGCCCCAGTAG	GTGCCAGCAAGATCCAATCTAGAGGAGCCCCCCAGGCCCCAGTAG
NUP98	10	5'	TACAACCAGTGGCGGGCTCTTTG	GTGCCAGCAAGATCCAATCTAGATACAACCAGTGGCGGGCTCTTTG
NUP98	11	5'	GCTTGGTGCAGGATTTGGAACAG	GTGCCAGCAAGATCCAATCTAGAGCTTGGTGCAGGATTTGGAACAG
NUP98	13	5'	GATGTCAGACCCTAAGAAGAAGGAAGAG	GTGCCAGCAAGATCCAATCTAGAGATGTCAGACCCTAAGAAGAAGGAAGAG

NUP98	14	5'	AGCCAATGGAGCATTCATGCCCAA	GTGCCAGCAAGATCCAATCTAGAAGCCAATGGAGCATTCATGCCCAA
NUP98	9	5'	GGATTTGGTGCTGTTGGTTCG	GTGCCAGCAAGATCCAATCTAGAGGATTTGGTGCTGTTGGTTCG
OFD1	22	5'	GCAAGACCAGGAGTCGGCAGATAAG	GTGCCAGCAAGATCCAATCTAGAGCAAGACCAGGAGTCGGCAGATAAG
P2RY8	1	5'	CTTCTGCCGCTGCTTCTGCACAG	GTGCCAGCAAGATCCAATCTAGACTTCTGCCGCTGCTTCTGCACAG
PAG1	8	5'	AGACCCCACTCTCACAGAAGAAGAG	GTGCCAGCAAGATCCAATCTAGAAGACCCCACTCTCACAGAAGAAGAG
PAX5	4	5'	CAGCTTCCAGTCACAGCATAGGCT	GTGCCAGCAAGATCCAATCTAGACAGCTTCCAGTCACAGCATAGGCT
PCM1	26	5'	CCTAGTGAGAGCCTTGCTACTACTGATGAT	GTGCCAGCAAGATCCAATCTAGACCTAGTGAGAGCCTTGCTACTACTGATGAT
PCM1	36	5'	ACTGAATCTCCAGTGTTAGTGAATGACTAT	GTGCCAGCAAGATCCAATCTAGAACTGAATCTCCAGTGTTAGTGAATGACTAT
PDE4DIP	19	5'	GATGTCAGCATACCCAGATCCACATTAG	GTGCCAGCAAGATCCAATCTAGAGATGTCAGCATACCCAGATCCACATTAG
PICALM	16	5'	ACCGCTTGGAATGCTGCAACAATG	GTGCCAGCAAGATCCAATCTAGAACCGCTTGGAATGCTGCAACAATG
PICALM	19	5'	CTTTGGCCCTGTATCAGGAGCACAG	GTGCCAGCAAGATCCAATCTAGACTTTGGCCCTGTATCAGGAGCACAG
PICALM	18	5'	CCAACAGGCATGATAGGATATGGAATT	GTGCCAGCAAGATCCAATCTAGACCAACAGGCATGATAGGATATGGAATT
PICALM	8	5'	AAATCAAAGATTCTACAGCTGCAAGCAG	GTGCCAGCAAGATCCAATCTAGAAAATCAAAGATTCTACAGCTGCAAGCAG
PICALM	9	5'	GAACAGGCACGTTTGAAAGCTTTAAAG	GTGCCAGCAAGATCCAATCTAGAGAACAGGCACGTTTGAAAGCTTTAAAG
PICALM	10	5'	CATATTTTCTACCCCTAGTTCTTCTAACAG	GTGCCAGCAAGATCCAATCTAGACATATTTTCTACCCCTAGTTCTTCTAACAG
PICALM	11	5'	CTTCTCAGGTAGCAAGTACATGGGGAG	GTGCCAGCAAGATCCAATCTAGACTTCTCAGGTAGCAAGTACATGGGGAG
PICALM	12	5'	CTAGGACACCTACTCATGAAATGTTTGTTG	GTGCCAGCAAGATCCAATCTAGACTAGGACACCTACTCATGAAATGTTTGTTG
PICALM	13	5'	AATCTACAAATGTTATTGTAGATTCTGGGG	GTGCCAGCAAGATCCAATCTAGAAATCTACAAATGTTATTGTAGATTCTGGGG
PICALM	14	5'	ATCTTTAGCCAACCTTGTGGGCA	GTGCCAGCAAGATCCAATCTAGAATCTTTAGCCAACCTTGTGGGCA
PICALM	15	5'	GCATCGGAAATGGAACCACTAAGAA	GTGCCAGCAAGATCCAATCTAGAGCATCGGAAATGGAACCACTAAGAA
ZBTB16	3	5'	CTGAGAATGCACTTACTGGCTCATTCAG	GTGCCAGCAAGATCCAATCTAGACTGAGAATGCACTTACTGGCTCATTCAG
ZBTB16	4	5'	GGAGACACACAGGCAGACCCATACTG	GTGCCAGCAAGATCCAATCTAGAGGAGACACACAGGCAGACCCATACTG
RANBP2	18	5'	ACATTTCATGGGGCTCCACTAACAG	GTGCCAGCAAGATCCAATCTAGAACATTTCATGGGGCTCCACTAACAG
RCSD1	3	5'	CTGGGCCAGAATGGTGAGGAG	GTGCCAGCAAGATCCAATCTAGACTGGGCCAGAATGGTGAGGAG
RCSD1	2	5'	AGGCGGCTGCAGCCAAGGAG	GTGCCAGCAAGATCCAATCTAGAAGGCGGCTGCAGCCAAGGAG
RUNX1	8	5'	TCTCTGCAGAACTTTCCAGTCGACTCTCAA	GTGCCAGCAAGATCCAATCTAGATCTCTGCAGAACTTTCCAGTCGACTCTCAA
SET	7	5'	TGATGAAGGGGAGGAAGGAGAGGAA	GTGCCAGCAAGATCCAATCTAGATGATGAAGGGGAGGAAGGAGAGGAA
SET	7	5'	ATGAAGGGGAGGAAGGAGAGTCG	GTGCCAGCAAGATCCAATCTAGAATGAAGGGGAGGAAGGAGAGTCG
SFPQ	9	5'	GGTTCCATGATGGGAAGTGACATG	GTGCCAGCAAGATCCAATCTAGAGGTTCCATGATGGGAAGTGACATG
SNX2	3	5'	CGTGATCTTTGATAGATCCAGGGAAGAG	GTGCCAGCAAGATCCAATCTAGACGTGATCTTTGATAGATCCAGGGAAGAG
SPAG9	26	5'	ACATTGAGCCTTATGTAAGCAAAATGTTAG	GTGCCAGCAAGATCCAATCTAGAACATTGAGCCTTATGTAAGCAAAATGTTAG
SPTBN1	3	5'	GACCATCATCCTGCGCTTCCAG	GTGCCAGCAAGATCCAATCTAGAGACCATCATCCTGCGCTTCCAG

SSBP2	16	5'	AATTTCTTAAATCCTTTTCAGAGTGAGAGT	GTGCCAGCAAGATCCAATCTAGAAATTTCTTAAATCCTTTTCAGAGTGAGAGT
SSBP2	6	5'	GCCCCCATTGAGGATACCTAATCAG	GTGCCAGCAAGATCCAATCTAGAGCCCCCATTGAGGATACCTAATCAG
SSBP2	8	5'	GGAATGGTGCCCTTAGGACCACAG	GTGCCAGCAAGATCCAATCTAGAGGAATGGTGCCCTTAGGACCACAG
SSBP2	10	5'	CCAAACCCAACAAATGCCAATTCA	GTGCCAGCAAGATCCAATCTAGACCAAACCCAACAAATGCCAATTCA
SSBP2	5	5'	GTCCTGTACCACCAGGGTTCTTTCAG	GTGCCAGCAAGATCCAATCTAGAGTCCTGTACCACCAGGGTTCTTTCAG
STMN1	2	5'	TGTCTTCTATTCACCATGGCTTCTTCTG	GTGCCAGCAAGATCCAATCTAGATGTCTTCTATTCACCATGGCTTCTTCTG
STRN	6	5'	GAGAAAAGCGTCATTGATACTTCAACA	GTGCCAGCAAGATCCAATCTAGAGAGAAAAGCGTCATTGATACTTCAACA
STRN3	9	5'	CATGAAGGTGCAAGAGCAGAGGAAG	GTGCCAGCAAGATCCAATCTAGACATGAAGGTGCAAGAGCAGAGGAAG
STRN3	8	5'	GAAAGGGGAAGAAAGGGGTGAAGA	GTGCCAGCAAGATCCAATCTAGAGAAAGGGGAAGAAAGGGGTGAAGA
TCF7	2	5'	CCCAGCCGCAGCCCCCGCTG	GTGCCAGCAAGATCCAATCTAGACCCAGCCGCAGCCCCCGCTG
TCF7	3	5'	GCGGACATCAGCCAGAAGCAAG	GTGCCAGCAAGATCCAATCTAGAGCGGACATCAGCCAGAAGCAAG
TERF2	8	5'	AGAGGATGAACTGTTTCAAGTTCAGG	GTGCCAGCAAGATCCAATCTAGAAGAGGATGAACTGTTTCAAGTTCAGG
TPM3	8	5'	TGGAAAAGACAATTGATGACCTGGAAG	GTGCCAGCAAGATCCAATCTAGATGGAAAAGACAATTGATGACCTGGAAG
TPR	39	5'	CAGGATTCTCAAGATTCCATTGGAGAA	GTGCCAGCAAGATCCAATCTAGACAGGATTCTCAAGATTCCATTGGAGAA
TPR	22	5'	TTAGAAGAATCCCTGAACAAGGAAAAACAG	GTGCCAGCAAGATCCAATCTAGATTAGAAGAATCCCTGAACAAGGAAAAACAG
TRIM24	9	5'	CCAGCAACCTTCCATCTCTCATCAG	GTGCCAGCAAGATCCAATCTAGACCAGCAACCTTCCATCTCTCATCAG
TRIM24	10	5'	TTCTTGGCTCAACAAGCCATAAAACAG	GTGCCAGCAAGATCCAATCTAGATTCTTGGCTCAACAAGCCATAAAACAG
TRIM24	11	5'	GGTCTTATAATCTTCCCTCTCTTCCGGAT	GTGCCAGCAAGATCCAATCTAGAGGTCTTATAATCTTCCCTCTCTTCCGGAT
XPO1	15	5'	TAACAAGCTGTTCGAATTCATGCATG	GTGCCAGCAAGATCCAATCTAGATAACAAGCTGTTCGAATTCATGCATG
XPO1	16	5'	ACTATTATTTGTGATCTTCAGCCTCAACAG	GTGCCAGCAAGATCCAATCTAGAACTATTATTTGTGATCTTCAGCCTCAACAG
XPO1	17	5'	GATAGTATAATCCAGCAGGCAACCAAA	GTGCCAGCAAGATCCAATCTAGAGATAGTATAATCCAGCAGGCAACCAAA
XPO1	18	5'	AAAATATTTCTGCAGCTATCCAAGCTAATG	GTGCCAGCAAGATCCAATCTAGAAAAATATTTCTGCAGCTATCCAAGCTAATG
XPO1	19	5'	CCGATCCAATGATCCACAGATG	GTGCCAGCAAGATCCAATCTAGACCGATCCAATGATCCACAGATG
XPO1	20	5'	TTTGAATGCACATTGAATATGATAAATAAG	GTGCCAGCAAGATCCAATCTAGATTTGAATGCACATTGAATATGATAAATAAG
XPO1	21	5'	ATACTATGAGGAATGTCGCAGATACGG	GTGCCAGCAAGATCCAATCTAGAATACTATGAGGAATGTCGCAGATACGG
XPO1	22	5'	GTTGTGACAGACACTTCACATACTGCTG	GTGCCAGCAAGATCCAATCTAGAGTTGTGACAGACACTTCACATACTGCTG
XPO1	23	5'	GGCCTTCCCTCACCTACAAGA	GTGCCAGCAAGATCCAATCTAGAGGCCTTCCCTCACCTACAAGA
XPO1	24	5'	CATTTAAGAGATTTCCTAGTTCAAATAAAG	GTGCCAGCAAGATCCAATCTAGACATTTAAGAGATTTCCTAGTTCAAATAAAG
ZC3HAV1	12	5'	ATGAAACTCATGAAAACAAATACGGAAAAG	GTGCCAGCAAGATCCAATCTAGAATGAAACTCATGAAAACAAATACGGAAAAG
ZEB2	10	5'	CCTTCTGCGACATAAATACGAACACACAG	GTGCCAGCAAGATCCAATCTAGACCTTCTGCGACATAAATACGAACACACAG
ZMIZ1	18	5'	CCGCAGAGCACTGTATCACGAAAA	GTGCCAGCAAGATCCAATCTAGACCGCAGAGCACTGTATCACGAAAA
ZMYM2	16	5'	ATTCCTGTTCCTACTACAGTTCCTGTTCCT	GTGCCAGCAAGATCCAATCTAGAATTCCTGTTCCTACTACAGTTCCTGTTCCT

SEPTIN5	**3**	**3'**	**GACATTGACAAGCAGTACGTGGGC**	**GACATTGACAAGCAGTACGTGGGCTCCAACCCTTAGGGAACCC**
SEPTIN9	**2**	**3'**	**GAGGCACGCGGACCTCCAGTGGC**	**GAGGCACGCGGACCTCCAGTGGCTCCAACCCTTAGGGAACCC**
SEPTIN9	**3**	**3'**	**CCTTGAAAAGATCTTTTGAGGTCGAGG**	**CCTTGAAAAGATCTTTTGAGGTCGAGGTCCAACCCTTAGGGAACCC**
ABL1	**2**	**3'**	**AAGCCCTTCAGCGGCCAGTAGC**	**AAGCCCTTCAGCGGCCAGTAGCTCCAACCCTTAGGGAACCC**
ABL1	**3**	**3'**	**GTGAAAAGCTCCGGGTCTTAGGC**	**GTGAAAAGCTCCGGGTCTTAGGCTCCAACCCTTAGGGAACCC**
ABL1	**4**	**3'**	**CTCTACGTCTCCTCCGAGAGCCG**	**CTCTACGTCTCCTCCGAGAGCCGTCCAACCCTTAGGGAACCC**
ABL2	**5**	**3'**	**GTGTATGTGACTGCTGAGAGCCGCT**	**GTGTATGTGACTGCTGAGAGCCGCTTCCAACCCTTAGGGAACCC**
ABL2	**3**	**3'**	**AAGCTTTGCATCGTCCCTATGGTTG**	**AAGCTTTGCATCGTCCCTATGGTTGTCCAACCCTTAGGGAACCC**
ABL2	**2**	**3'**	**ATCACTTTGCCAGCTGTGTGGAGGAT**	**ATCACTTTGCCAGCTGTGTGGAGGATTCCAACCCTTAGGGAACCC**
ADD3	**14**	**3'**	**ATGCTGAGCAGGAATTACTCTCAGATGACG**	**ATGCTGAGCAGGAATTACTCTCAGATGACGTCCAACCCTTAGGGAACCC**
ADD3	**15**	**3'**	**AAAACCATGAGCTGTTTTCCAAGAGCTTCA**	**AAAACCATGAGCTGTTTTCCAAGAGCTTCATCCAACCCTTAGGGAACCC**
MLLT10	**10**	**3'**	**ACTTATACAAGCACTAGCAACAACTCTATA**	**ACTTATACAAGCACTAGCAACAACTCTATATCCAACCCTTAGGGAACCC**
MLLT10	**16**	**3'**	**GCAAATACTCTATCTGGATCTTCTCTC**	**GCAAATACTCTATCTGGATCTTCTCTCTCCAACCCTTAGGGAACCC**
MLLT6	**8**	**3'**	**AGTCGAAAGGACAAAGAACGCCTTAAG**	**AGTCGAAAGGACAAAGAACGCCTTAAGTCCAACCCTTAGGGAACCC**
MLLT6	**9**	**3'**	**GTCTCCTCCTCGGCTTCCTCTTC**	**GTCTCCTCCTCGGCTTCCTCTTCTCCAACCCTTAGGGAACCC**
MLLT6	**12**	**3'**	**GTGTTTTCTCTGGCTGGCTCTACC**	**GTGTTTTCTCTGGCTGGCTCTACCTCCAACCCTTAGGGAACCC**
MLLT11	**2**	**3'**	**GAAGCTATGAGGGACCCTGTGAG**	**GAAGCTATGAGGGACCCTGTGAGTCCAACCCTTAGGGAACCC**
AFF1	**4**	**3'**	**CAGACCTACTCCAATGAAGTCCATTG**	**CAGACCTACTCCAATGAAGTCCATTGTCCAACCCTTAGGGAACCC**
AFF1	**5**	**3'**	**GAAATGACCCATTCATGGCCGCC**	**GAAATGACCCATTCATGGCCGCCTCCAACCCTTAGGGAACCC**
AFF1	**6**	**3'**	**GACTCTCAGCATGTCAGTTCTGTAAC**	**GACTCTCAGCATGTCAGTTCTGTAACTCCAACCCTTAGGGAACCC**
AFF1	**7**	**3'**	**CCTGAGCCTCCAACAACAAACAAATG**	**CCTGAGCCTCCAACAACAAACAAATGTCCAACCCTTAGGGAACCC**
AFDN	**2**	**3'**	**GATTTGGAGTTCCATGGAGTGATGAG**	**GATTTGGAGTTCCATGGAGTGATGAGTCCAACCCTTAGGGAACCC**
MLLT3	**5**	**3'**	**GACCCTAATAGGAGTATTCATACCAGC**	**GACCCTAATAGGAGTATTCATACCAGCTCCAACCCTTAGGGAACCC**
MLLT3	**6**	**3'**	**TCTGAACAACCCAGTCCTGCCAG**	**TCTGAACAACCCAGTCCTGCCAGTCCAACCCTTAGGGAACCC**
MLLT3	**10**	**3'**	**GCATACCTAGATGAACTGGTAGAGC**	**GCATACCTAGATGAACTGGTAGAGCTCCAACCCTTAGGGAACCC**
MLLT3	**4**	**3'**	**GAAGAACCTAGGAAAGTCCGCTTTG**	**GAAGAACCTAGGAAAGTCCGCTTTGTCCAACCCTTAGGGAACCC**
MLLT3	**9**	**3'**	**ATTCTTGAAGTGAAAAGTCCAATAAAGCAAA**	**ATTCTTGAAGTGAAAAGTCCAATAAAGCAAATCCAACCCTTAGGGAACCC**
AFF3	**5**	**3'**	**GAGAGTAGATCTGGAGAAACCAACAG**	**GAGAGTAGATCTGGAGAAACCAACAGTCCAACCCTTAGGGAACCC**
AFF3	**6**	**3'**	**GAGATGACCTGGCTTCCACCACT**	**GAGATGACCTGGCTTCCACCACTTCCAACCCTTAGGGAACCC**
AFF3	**10**	**3'**	**CAGGCAGCTCAGAGAACGGCTCT**	**CAGGCAGCTCAGAGAACGGCTCTTCCAACCCTTAGGGAACCC**
AFF3	**9**	**3'**	**AGAAAGGTGATGCAGAGCCAGAGAGTCC**	**AGAAAGGTGATGCAGAGCCAGAGAGTCCTCCAACCCTTAGGGAACCC**
AFF4	**6**	**3'**	**GAGTCTCAGCAGTCCAATTTTGGC**	**GAGTCTCAGCAGTCCAATTTTGGCTCCAACCCTTAGGGAACCC**

AFF4	4	3'	GCATCAGCTTCTGGTGATGTGAGC	GCATCAGCTTCTGGTGATGTGAGCTCCAACCCTTAGGGAACCC
AFF4	5	3'	GAGATGACGCATTCATGGCCTCC	GAGATGACGCATTCATGGCCTCCTCCAACCCTTAGGGAACCC
ARHGEF12	11	3'	GATGGAGCTGTAGTTACACCCTCC	GATGGAGCTGTAGTTACACCCTCCTCCAACCCTTAGGGAACCC
ARHGEF12	12	3'	AGTCCCAAGAGTGGCCCAAAAGAG	AGTCCCAAGAGTGGCCCAAAAGAGTCCAACCCTTAGGGAACCC
ARHGEF12	13	3'	GACACTCAATCACTTGTCGGAAGTC	GACACTCAATCACTTGTCGGAAGTCTCCAACCCTTAGGGAACCC
ARHGEF17	2	3'	GACATGCGGAAGCACGTGGCCAT	GACATGCGGAAGCACGTGGCCATTCCAACCCTTAGGGAACCC
ARHGEF17	3	3'	GGCTACATGCAGCCGCTGAAGCA	GGCTACATGCAGCCGCTGAAGCATCCAACCCTTAGGGAACCC
ARHGEF17	4	3'	TTCTCCAAGGATGTCCTAGTAAACATC	TTCTCCAAGGATGTCCTAGTAAACATCTCCAACCCTTAGGGAACCC
ARHGEF17	5	3'	CAAAGCATGCGTGAGAACAAGGAG	CAAAGCATGCGTGAGAACAAGGAGTCCAACCCTTAGGGAACCC
ARNT	2	3'	AAATGACATCAGATGTACCATCACTGGG	AAATGACATCAGATGTACCATCACTGGGTCCAACCCTTAGGGAACCC
C2CD3	13	3'	CCAGAAGTCATTGGATCTGTGTCAC	CCAGAAGTCATTGGATCTGTGTCACTCCAACCCTTAGGGAACCC
C2CD3	14	3'	GTAACCATGGAGCTTATTACAGATAACAAA	GTAACCATGGAGCTTATTACAGATAACAAATCCAACCCTTAGGGAACCC
C2CD3	15	3'	GTGATTCCTGTCTCTCTGTCTTCC	GTGATTCCTGTCTCTCTGTCTTCCTCCAACCCTTAGGGAACCC
C2CD3	17	3'	GCAGAGGACCGAGGAAATGGACT	GCAGAGGACCGAGGAAATGGACTTCCAACCCTTAGGGAACCC
NUP214	17	3'	CTTCATGGAGATATAAGTAGCCTGAA	CTTCATGGAGATATAAGTAGCCTGAATCCAACCCTTAGGGAACCC
NUP214	18	3'	ATTCGGCGCCTTCATCAGTATGTG	ATTCGGCGCCTTCATCAGTATGTGTCCAACCCTTAGGGAACCC
KNL1	12	3'	ATTTTTGATCACCATACTGAAGAGGATATAG	ATTTTTGATCACCATACTGAAGAGGATATAGTCCAACCCTTAGGGAACCC
CBL	10	3'	GTGGAACGGCCGCCTTCTCCATT	GTGGAACGGCCGCCTTCTCCATTTCCAACCCTTAGGGAACCC
CIP2A	17	3'	GCACGGACACTTGCTAGTATGTTG	GCACGGACACTTGCTAGTATGTTGTCCAACCCTTAGGGAACCC
CRLF2	2	3'	GCATGGGGCGGCTGGTTCTGCTG	GCATGGGGCGGCTGGTTCTGCTGTCCAACCCTTAGGGAACCC
CSF1R	12	3'	AAGCCCAAGTACCAGGTCCGCT	AAGCCCAAGTACCAGGTCCGCTTCCAACCCTTAGGGAACCC
DCPS	2	3'	GTGAATGAGGCCTCTGGGGATGG	GTGAATGAGGCCTCTGGGGATGGTCCAACCCTTAGGGAACCC
DDX10	6	3'	TTCTTGATGAAGCAGATAGAATCTTGG	TTCTTGATGAAGCAGATAGAATCTTGGTCCAACCCTTAGGGAACCC
DDX10	7	3'	CACCCCTGCCACTTTGGAACAGA	CACCCCTGCCACTTTGGAACAGATCCAACCCTTAGGGAACCC
ELL	2	3'	GATTCTGTTTCACTGAGGCCATCTATC	GATTCTGTTTCACTGAGGCCATCTATCTCCAACCCTTAGGGAACCC
ELL	3	3'	CACATCTCCATCCCCCAGCCTGA	CACATCTCCATCCCCCAGCCTGATCCAACCCTTAGGGAACCC
ELL	6	3'	GTGGCCAACATGAGTGCTAAGGAC	GTGGCCAACATGAGTGCTAAGGACTCCAACCCTTAGGGAACCC
MLLT1	2	3'	TGCACCGTCCAGGTGAGGTTAGA	TGCACCGTCCAGGTGAGGTTAGATCCAACCCTTAGGGAACCC
MLLT1	7	3'	TCTGCCCAGTCAAGCCCGTCCAA	TCTGCCCAGTCAAGCCCGTCCAATCCAACCCTTAGGGAACCC
MLLT1	4	3'	GAGGAGCCGAGGAAGGTCTGCTT	GAGGAGCCGAGGAAGGTCTGCTTTCCAACCCTTAGGGAACCC
MLLT1	5	3'	GTGATGGTAATGCCCGAAGGAGC	GTGATGGTAATGCCCGAAGGAGCTCCAACCCTTAGGGAACCC
MLLT1	6	3'	GACGCCAACAAGGAGAGCAGCAA	GACGCCAACAAGGAGAGCAGCAATCCAACCCTTAGGGAACCC

EPS15	2	3'	TTATCAAGTGGGAATCCTGTATATGAAAAAT	TTATCAAGTGGGAATCCTGTATATGAAAAATTCCAACCCTTAGGGAACCC
EPS15	6	3'	CATGATACCAGTAGTCCTTTGCTAATC	CATGATACCAGTAGTCCTTTGCTAATCTCCAACCCTTAGGGAACCC
FER	14	3'	GACAAGAAATGGATTCTCAGTCATGAAGAT	GACAAGAAATGGATTCTCAGTCATGAAGATTCCAACCCTTAGGGAACCC
FGFR1	10	3'	GTGTCTGCTGACTCCAGTGCATCC	GTGTCTGCTGACTCCAGTGCATCCTCCAACCCTTAGGGAACCC
FLT3	14	3'	CAATTTAGGTATGAAAGCCAGCTACAGATG	CAATTTAGGTATGAAAGCCAGCTACAGATGTCCAACCCTTAGGGAACCC
FOXO3	3	3'	AACTCCATCCGGCACAACCTGTC	AACTCCATCCGGCACAACCTGTCTCCAACCCTTAGGGAACCC
FOXO4	2	3'	AACTCGATCCGCCACAACCTG	AACTCGATCCGCCACAACCTGTCCAACCCTTAGGGAACCC
ARHGAP26	19	3'	ATATTTAACACCGTGCCCGATATGCC	ATATTTAACACCGTGCCCGATATGCCTCCAACCCTTAGGGAACCC
HOXA9	1	3'	TTGATAGAGAAAAACAACCCAGCGAAG	TTGATAGAGAAAAACAACCCAGCGAAGTCCAACCCTTAGGGAACCC
HOXA9	2	3'	ATAACCCAGCAGCCAACTGGCTTC	ATAACCCAGCAGCCAACTGGCTTCTCCAACCCTTAGGGAACCC
JAK2	9	3'	GAAATTGAACTTAGCTCATTAAGGGAAGCT	GAAATTGAACTTAGCTCATTAAGGGAAGCTTCCAACCCTTAGGGAACCC
JAK2	17	3'	TTCTTCAGGAGAGAATACCATGGGTACC	TTCTTCAGGAGAGAATACCATGGGTACCTCCAACCCTTAGGGAACCC
JAK2	19	3'	ATTATGAACTATTAACAGAAAATGACATGT	ATTATGAACTATTAACAGAAAATGACATGTTCCAACCCTTAGGGAACCC
JAK2	11	3'	CGAGAAAATGTCATTGAATATAAACACTGT	CGAGAAAATGTCATTGAATATAAACACTGTTCCAACCCTTAGGGAACCC
JAK2	15	3'	ATATTCTGGTTCAGGAGTTTGTAAAATTTG	ATATTCTGGTTCAGGAGTTTGTAAAATTTGTCCAACCCTTAGGGAACCC
JAK2	18	3'	AAGCTACAATTTTATGAAGATAGGCATCAG	AAGCTACAATTTTATGAAGATAGGCATCAGTCCAACCCTTAGGGAACCC
JAK2	13	3'	AATGAAAGCCTTGGCCAAGGC	AATGAAAGCCTTGGCCAAGGCTCCAACCCTTAGGGAACCC
KDM5A	27	3'	GATGACAGCATGGAAGAGAAACCACTA	GATGACAGCATGGAAGAGAAACCACTATCCAACCCTTAGGGAACCC
LYN	7	3'	AGCAGGCAGATGGCTTGTGCA	AGCAGGCAGATGGCTTGTGCATCCAACCCTTAGGGAACCC
MAML2	2	3'	CTCCAGGGTTCCTTGAAAAGAAAACAG	CTCCAGGGTTCCTTGAAAAGAAAACAGTCCAACCCTTAGGGAACCC
MAML2	3	3'	GATCAACACTCTGTGGTAGGCCAG	GATCAACACTCTGTGGTAGGCCAGTCCAACCCTTAGGGAACCC
MAPRE1	2	3'	AAGATGGCAGTGAACGTATACTCAACG	AAGATGGCAGTGAACGTATACTCAACGTCCAACCCTTAGGGAACCC
MAPRE1	4	3'	ATAATTCCTGTGGACAAATTAGTAAAAGGA	ATAATTCCTGTGGACAAATTAGTAAAAGGATCCAACCCTTAGGGAACCC
MAPRE1	6	3'	GTCAACGTATTGAAACTTACTGTTGAAGAC	GTCAACGTATTGAAACTTACTGTTGAAGACTCCAACCCTTAGGGAACCC
KMT2A	3	3'	GATGAGCAATTCTTAGGTTTTGGCTCA	GATGAGCAATTCTTAGGTTTTGGCTCATCCAACCCTTAGGGAACCC
MLLT10	4	3'	AGATGTGAACTTTGTCCCCATAAGGAT	AGATGTGAACTTTGTCCCCATAAGGATTCCAACCCTTAGGGAACCC
MLLT10	6	3'	ACTTGCTACATTTGTGATGAACAAGGAAG	ACTTGCTACATTTGTGATGAACAAGGAAGTCCAACCCTTAGGGAACCC
MLLT10	9	3'	AAATATAAAGAGAAGGACAAACACAAACAGA	AAATATAAAGAGAAGGACAAACACAAACAGATCCAACCCTTAGGGAACCC
MLLT10	3	3'	CTTGCTATGGCATTGTTCAAGTACCC	CTTGCTATGGCATTGTTCAAGTACCCTCCAACCCTTAGGGAACCC
MLLT10	5	3'	GTTGGGCCCATGTGGTTTGTG	GTTGGGCCCATGTGGTTTGTGTCCAACCCTTAGGGAACCC
MLLT10	7	3'	CGCTCAGTTTGCCGGACTGC	CGCTCAGTTTGCCGGACTGCTCCAACCCTTAGGGAACCC
MLLT10	8	3'	AAAAAGAGCAAACGGGGATCTAATAGGT	AAAAAGAGCAAACGGGGATCTAATAGGTTCCAACCCTTAGGGAACCC

MLLT10	11	3'	GCAGTTTTTCAGGAACTCCAGGC	GCAGTTTTTCAGGAACTCCAGGCTCCAACCCTTAGGGAACCC
MLLT10	12	3'	AAATTTCCATGCAGTATCGGCATGAT	AAATTTCCATGCAGTATCGGCATGATTCCAACCCTTAGGGAACCC
MLLT10	13	3'	CGTTCTCAGAGTTGCTGAATGCAATA	CGTTCTCAGAGTTGCTGAATGCAATATCCAACCCTTAGGGAACCC
MLLT10	14	3'	ACAGAGGTGACAGTTCTACACTAACAAAGC	ACAGAGGTGACAGTTCTACACTAACAAAGCTCCAACCCTTAGGGAACCC
MLLT10	15	3'	GTATTTATAACAGCAATGATGTAGCAGTAT	GTATTTATAACAGCAATGATGTAGCAGTATTCCAACCCTTAGGGAACCC
MLLT10	17	3'	ATCAAGATCTTGGAGACAATAGCCGC	ATCAAGATCTTGGAGACAATAGCCGCTCCAACCCTTAGGGAACCC
MLLT10	18	3'	ATCCCCTGTAAGCAGCTTACAGATTCG	ATCCCCTGTAAGCAGCTTACAGATTCGTCCAACCCTTAGGGAACCC
MLLT10	19	3'	TTTTAGGAATGCTGAAGTCATTACACCAAC	TTTTAGGAATGCTGAAGTCATTACACCAACTCCAACCCTTAGGGAACCC
MLLT10	20	3'	CTCCTACTACTGATTCCTTGAACAGCAGTA	CTCCTACTACTGATTCCTTGAACAGCAGTATCCAACCCTTAGGGAACCC
MLLT10	21	3'	GACTTAACCTCCAGTGGACAAAGTACCA	GACTTAACCTCCAGTGGACAAAGTACCATCCAACCCTTAGGGAACCC
MLLT10	22	3'	TGCCTCAGGACTAGGATTACTTTCTGAC	TGCCTCAGGACTAGGATTACTTTCTGACTCCAACCCTTAGGGAACCC
MLLT10	23	3'	GTACACAGGCACCCCCACTTCAC	GTACACAGGCACCCCCACTTCACTCCAACCCTTAGGGAACCC
NCOA2	13	3'	CTTTTAATAACCCACGACCAGGGC	CTTTTAATAACCCACGACCAGGGCTCCAACCCTTAGGGAACCC
NCOA2	14	3'	GAATGATTGGTAACAGTGCTTCTCGGCCTA	GAATGATTGGTAACAGTGCTTCTCGGCCTATCCAACCCTTAGGGAACCC
NCOA2	15	3'	GGCCATCTGAATTAGAGATGAACATGGG	GGCCATCTGAATTAGAGATGAACATGGGTCCAACCCTTAGGGAACCC
NRIP3	2	3'	CAACCTCATAATATTCTGCAGAGGCG	CAACCTCATAATATTCTGCAGAGGCGTCCAACCCTTAGGGAACCC
NSD1	6	3'	CTGTGCGGTCAGAGAAGAAACGC	CTGTGCGGTCAGAGAAGAAACGCTCCAACCCTTAGGGAACCC
NTRK3	15	3'	ATGTGCAGCACATTAAGAGGAGAGACAT	ATGTGCAGCACATTAAGAGGAGAGACATTCCAACCCTTAGGGAACCC
NTRK3	14	3'	GTCCCGTGGCTGTCATCAGTGG	GTCCCGTGGCTGTCATCAGTGGTCCAACCCTTAGGGAACCC
PDGFRA	12	3'	CTGCCTTATGACTCAAGATGGGAGTTTC	CTGCCTTATGACTCAAGATGGGAGTTTCTCCAACCCTTAGGGAACCC
PDGFRB	11	3'	CCTTGCCCTTTAAGGTGGTGGTG	CCTTGCCCTTTAAGGTGGTGGTGTCCAACCCTTAGGGAACCC
PDGFRB	9	3'	TCCCTGTCCGAGTGCTGGAGC	TCCCTGTCCGAGTGCTGGAGCTCCAACCCTTAGGGAACCC
PSIP1	7	3'	GCAGAAAAACAAGTAGAAACTGAGGAGG	GCAGAAAAACAAGTAGAAACTGAGGAGGTCCAACCCTTAGGGAACCC
PTPRK	2	3'	GTGGCTGTACTTTTGATGATGGTCCAGG	GTGGCTGTACTTTTGATGATGGTCCAGGTCCAACCCTTAGGGAACCC
RAP1GDS1	2	3'	ATAATCTCAGTGATACCTTGAAGAAGCTG	ATAATCTCAGTGATACCTTGAAGAAGCTGTCCAACCCTTAGGGAACCC
RAP1GDS1	3	3'	ATACGGAAACAAGTGAAAAAATCCAAGC	ATACGGAAACAAGTGAAAAAATCCAAGCTCCAACCCTTAGGGAACCC
SETPIN6	2	3'	GGTGAAGGTTGCCGAACTGTCCC	GGTGAAGGTTGCCGAACTGTCCCTCCAACCCTTAGGGAACCC
SPI1	2	3'	CCATCAGAAGACCTGGTGCCCTAT	CCATCAGAAGACCTGGTGCCCTATTCCAACCCTTAGGGAACCC
SPI1	3	3'	ACCATTACTGGGACTTCCACCCC	ACCATTACTGGGACTTCCACCCCTCCAACCCTTAGGGAACCC
TET1	9	3'	GAAAAAAACCTTGAAGATAACTTACAGAGTT	GAAAAAAACCTTGAAGATAACTTACAGAGTTTCCAACCCTTAGGGAACCC
TOP1	8	3'	GATGGTAAATTGAAAAAACCCAAGAAT	GATGGTAAATTGAAAAAACCCAAGAATTCCAACCCTTAGGGAACCC

Table S3A. Biological characteristics of T acute lymphoblastic leukemia according to fusion transcripts status in adult patients

[a]All means are compared against the Negative group with Wilcoxon test or Student test depending on the Shapiro test result. All proportions are compared against the Negative group with Pearson's $\chi 2$ test or for small values with the Fisher exact test. Results with $p \leq 0.05$ are displayed in bold.

**RT-MLPA**			Positive		Negative	**All**
			60		256	316
**Fusion groups**	**MLLT10** (n = 22) 7 %	**KMT2A/SET** (n = 26) 8 %	**ABL1** (n = 10) 3 %	**Other** (n = 2) 1 %	81 %	
**Transcripts**	PICALM-MLLT10 (14)<br>DDX3X-MLLT10 (5)<br>NAP1L1-MLLT10 (2)<br>XPO1-MLLT10 (1)	SET-NUP214 (15)<br>KMT2A-AFDN (8)<br>KMT2A-MLLT1 (1)<br>KMT2A-ELL (1)<br>KMT2A-? (1)	NUP214-ABL1 (8)<br>BCR-ABL1 (1)<br>ETV6-ABL1 (1)	NUP98-RAP1GDS1 (1)<br>PCM1-FLT3 (1)		
**Population[a]**						
Age mean (range)	30.4 [19 - 44]	33.1 [21 - 63]	39.2 [19 - 61]	30.5 [27 - 34]	36.2 [19 - 78]	35.6 [19 - 78]
**Phenotype[a]**						
Immature	5 % (n = 1)	5 % (n = 1)	20 % (n = 1)	0	13 % (n = 27)	12 % (n = 30)
γδ-lineage	**80 % (n = 16)**	**86 % (n = 18)**	40 % (n = 2)	0	30 % (n = 60)	38 % (n = 96)
αβ-lineage	**15 % (n = 3)**	**10 % (n = 2)**	40 % (n = 2)	100 % (n = 1)	57 % (n = 116)	50 % (n = 124)
Not available	9 % (n = 2)	19 % (n = 5)	**50 % (n = 5)**	50 % (n = 1)	21 % (n = 53)	21 % (n = 66)
**Homeobox Genes Deregulation[a]**						
HOXA9	**91 % (n = 20)**	**100 % (n = 26)**	10 % (n = 1)	50 % (n = 1)	25 % (n = 64)	35 % (n = 112)
TLX1	0	0	30 % (n = 3)	0	20 % (n = 52)	17 % (n = 55)
TLX3	0	0	**50 % (n = 5)**	0	9 % (n = 23)	9 % (n = 28)
Negative	**9 % (n = 2)**	0	20 % (n = 2)	50 % (n = 1)	49 % (n = 126)	41 % (n = 131)

Table S3B. Biological characteristics of T acute lymphoblastic leukemia according to fusion transcripts status in pediatric patients

[a]All means are compared against the Negative group with Wilcoxon test or Student test depending on the Shapiro test result. All proportions are compared against the Negative group with Pearson's $\chi 2$ test or for small values with the Fisher exact test. Results with $p \leq 0.05$ are displayed in bold.

**RT-MLPA**					Positive	Negative	**All**
					44	162	206
**Fusion groups**	**MLLT10** (n = 15) 7 %	**KMT2A/SET** (n = 8) 4 %	**ABL1** (n = 12) 6 %	**ETS** (n = 6) 3 %	**Other** (n = 3) 1 %	79 %	
**Transcripts**	PICALM-MLLT10 (9)<br>DDX3X-MLLT10 (3)<br>NAP1L1-MLLT10 (2)<br>XPO1-MLLT10 (1)	KMT2A-ENL (3)<br>SET-NUP214 (3)<br>KMT2A-AFDN (2)	NUP214-ABL1 (11)<br>BCR-ABL1 (1)	ETV6-NCOA2 (3)<br>STMN1-SPI1 (2)<br>TCF7-SPI1 (1)	NUP98-RAP1GDS1 (2)<br>P2RY8-CRLF2 (1)		
**Population[a]**							
Age mean (range)	**13 [3 - 18]**	11.1 [8 - 17]	9.8 [4 - 16]	**4.5 [1 - 8]**	11 [8 - 13]	9.6 [1 - 18]	9.8 [1 - 18]
**Phenotype[a]**							
Immature	0	0	0	33 % (n = 1)	50 % (n = 1)	5 % (n = 7)	6 % (n = 9)
γδ-lineage	**92 % (n = 11)**	**67 % (n = 4)**	20 % (n = 2)	0	50 % (n = 1)	19 % (n = 25)	26 % (n = 43)
αβ-lineage	**8 % (n = 1)**	**33 % (n = 2)**	80 % (n = 8)	67 % (n = 2)	0	75 % (n = 98)	68 % (n = 111)
Not available	20 % (n = 3)	25 % (n = 2)	17 % (n = 2)	50 % (n = 3)	33 % (n = 1)	20 % (n = 32)	21 % (n = 43)
**Homeobox Genes Deregulation[a]**							
HOXA9	**100 % (n = 15)**	**100 % (n = 8)**	0	0	**67 % (n = 2)**	11 % (n = 18)	21 % (n = 43)
TLX1	0	0	0	0	0	5 % (n = 8)	4 % (n = 8)
TLX3	0	0	**92 % (n = 11)**	0	0	21 % (n = 34)	22 % (n = 45)
Negative	0	0	**8 % (n = 1)**	100 % (n = 6)	33 % (n = 1)	65 % (n = 105)	55 % (n = 113)

Table S4. 5-year EFS, CIR and OS in GRAALL and FRALLE treated patients according to fusion transcripts groups.

All subgroups characterisitics are compared with the negative group. All Results with p-value ≤0.05 are displayed with *. Values in bold represent trend.

	5y-Event Free Survival			5y-Cumulative Incidence of Relapse			5y-Overall Survival		
	HR	95%CI	p	HR	95%CI	p	HR	95%CI	p
ABL1 fusions (n=15)	**1.81**	**0.91-3.59**	**0.09**	1.43	0.52-3.9	0.49	**1.89**	**0.87-4.15**	**0.11**
KMT2A fusions (n=11)	0.75	0.28-2.05	0.58	0.83	0.26-2.64	0.75	0.83	0.26-2.63	0.75
SET fusions (n=8)	1.25	0.50-3.10	0.63	**1.92**	**0.76-4.85**	**0.17**	0.59	0.14-2.42	0.46
MLLT10 fusions (n=20)	**2.33**	**1.34-4.06**	**0.003***	1.77	0.84-3.74	0.13	**1.94**	**0.99-3.8**	**0.05**

3. Supplemental figures

Figure S1. Consort diagram

* Diagnosis peripheral blood or bone marrow samples

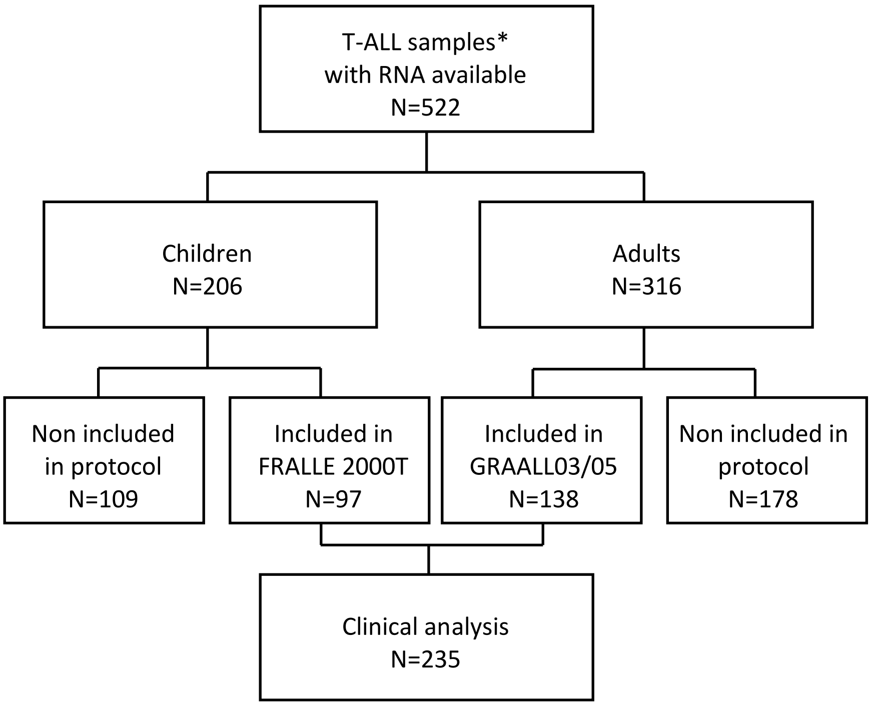

Figure S2. Fusion transcripts

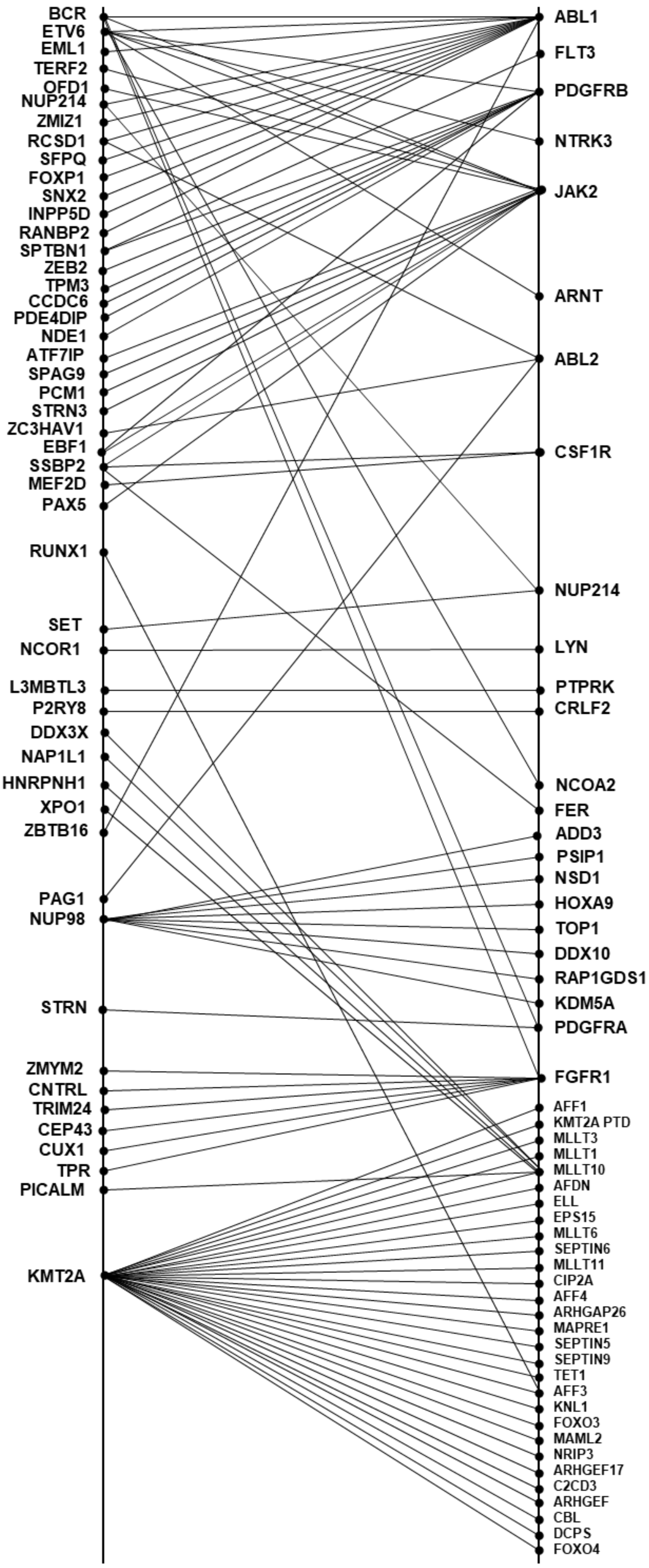

Figure S3. Event Free Survival (EFS)(A) and Overall survival (OS)(B) in GRAALL and FRALLE treated patients according to HOXA9 overexpression in T-ALL with fusion transcript.

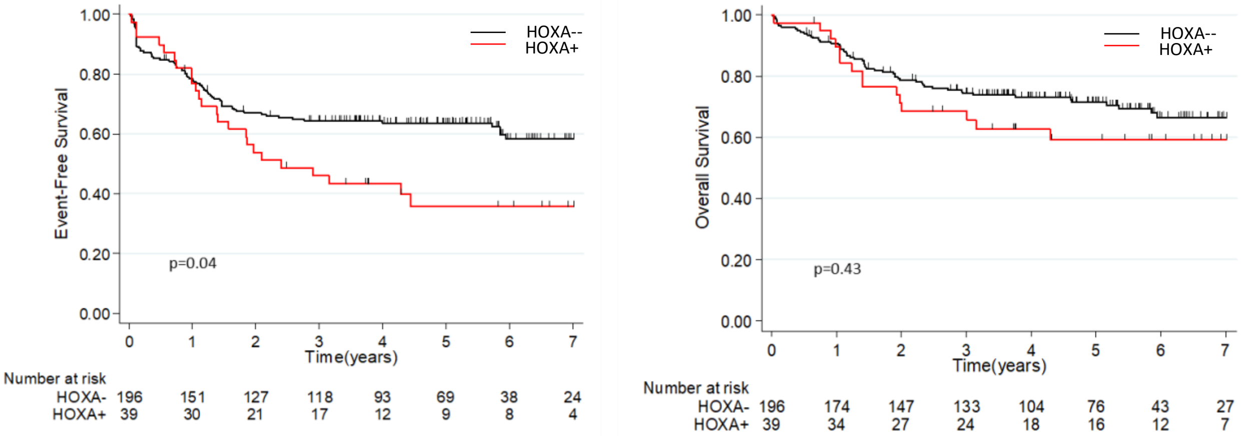

Figure S4. Event Free Survival (EFS)(A)(C) and Overall survival (OS)(B)(D) in GRAALL (A, B) and FRALLE 2000T (C, D) treated patients according to MLLT10 fusions status.

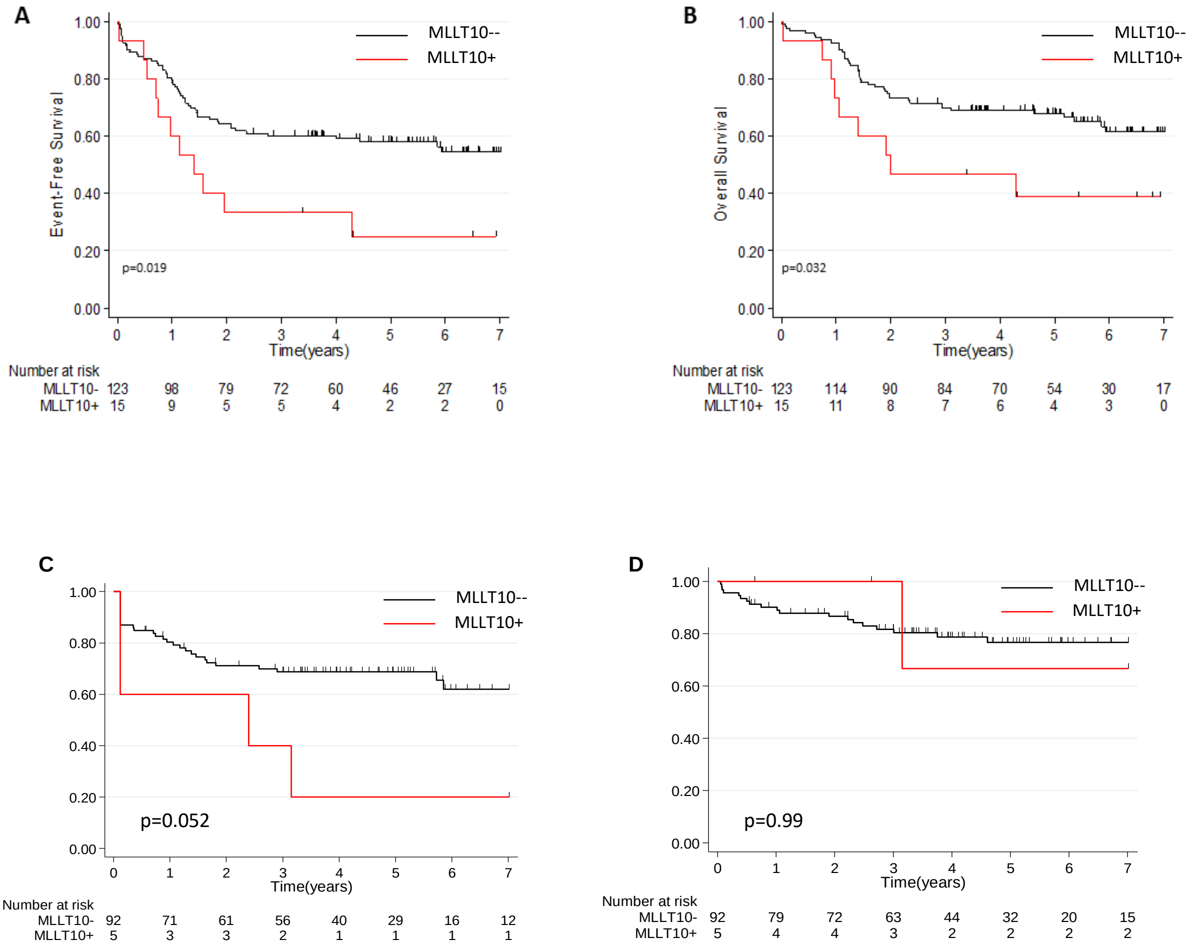

Supplement: Supplementary file 1 — Supplemental data [file 41408_2022_613_MOESM1_ESM.pdf]
